# A promoter trap in transgenic citrus mediates recognition of a broad spectrum of *Xanthomonas citri* pv. *citri* TALEs, including *in planta*‐evolved derivatives

**DOI:** 10.1111/pbi.14109

**Published:** 2023-07-08

**Authors:** Deepak Shantharaj, Gerald V. Minsavage, Vladimir Orbović, Gloria A. Moore, Danalyn R. Holmes, Patrick Römer, Diana M. Horvath, Thomas Lahaye, Jeffrey B. Jones

**Affiliations:** ^1^ Plant Pathology Department University of Florida Gainesville FL USA; ^2^ Citrus Research and Education Center University of Florida Lake Alfred FL USA; ^3^ Department of Horticultural Sciences University of Florida Gainesville FL USA; ^4^ Zentrum für Molekularbiologie der Pflanzen (ZMBP) Eberhard‐Karls‐Universität Tübingen Tübingen Germany; ^5^ Genetics, Department of Biology Ludwig‐Maximilians‐University Munich Martinsried Germany; ^6^ 2Blades Foundation Evanston IL USA; ^7^ Present address: Avicare+ Köthen Germany

**Keywords:** *Xanthomonas citri* subsp. *citri* (*Xcc*), transcriptional activator‐like effector (TALE) protein, executor‐type resistance (*R*) gene, AvrBs3, AvrGf1, AvrGf2

## Abstract

Citrus bacterial canker (CBC), caused by *Xanthomonas citri* subsp. *citri* (*Xcc*), causes dramatic losses to the citrus industry worldwide. Transcription activator‐like effectors (TALEs), which bind to effector binding elements (*EBEs*) in host promoters and activate transcription of downstream host genes, contribute significantly to *Xcc* virulence. The discovery of the biochemical context for the binding of TALEs to matching *EBE* motifs, an interaction commonly referred to as the TALE code, enabled the *in silico* prediction of *EBEs* for each TALE protein. Using the TALE code, we engineered a synthetic resistance (*R*) gene, called the *Xcc‐TALE‐trap*, in which 14 tandemly arranged *EBEs*, each capable of autonomously recognizing a particular *Xcc* TALE, drive the expression of *Xanthomonas avrGf2*, which encodes a bacterial effector that induces plant cell death. Analysis of a corresponding transgenic Duncan grapefruit showed that transcription of the cell death‐inducing executor gene, *avrGf2*, was strictly TALE‐dependent and could be activated by several different *Xcc* TALE proteins. Evaluation of *Xcc* strains from different continents showed that the *Xcc*‐*TALE*‐*trap* mediates resistance to this global panel of *Xcc* isolates. We also studied *in planta*‐evolved TALEs (eTALEs) with novel DNA‐binding domains and found that these eTALEs also activate the *Xcc‐TALE‐trap*, suggesting that the *Xcc‐TALE‐trap* is likely to confer durable resistance to *Xcc*. Finally, we show that the *Xcc‐TALE‐trap* confers resistance not only in laboratory infection assays but also in more agriculturally relevant field studies. In conclusion, transgenic plants containing the *Xcc‐TALE‐trap* offer a promising sustainable approach to control CBC.

## Introduction

The bacterial pathogen *Xanthomonas citri* pv. *citri* (*Xcc*) is the causal agent of citrus bacterial canker (CBC), a disease associated with defoliation, blemished fruit, premature fruit drop, twig dieback, and general tree decline, thereby causing severe economic losses to the citrus industry worldwide (Naqvi *et al*., 2022). The movement of *Xcc*‐infected propagating material, such as budwood, rootstock seedlings, or budding trees has repeatedly led to outbreaks of CBC in citrus growing areas previously unaffected by CBC. Given the known long‐distance routes of *Xcc* dissemination, it is evident that genetic resistance is the only option for sustainable control of CBC and that bactericides are not a sustainable means of preventing future CBC outbreaks.

Research on plant pathogenic xanthomonads that infect various host species has provided insights into the molecular basis of how this pathogen manipulates susceptible hosts to promote disease and how pathogen‐resistant plant genotypes either avoid manipulation by the pathogen or use immune receptors to recognize and combat the pathogen. Many xanthomonads inject transcriptional activator‐like effector (TALE) proteins into plant host cells to increase their virulence towards the host. TALE proteins bind to effector‐binding elements (*EBEs*) in host promoters and transcriptionally activate downstream host susceptibility (*S*) genes to favour *in planta* growth and/or spread of the pathogen (Teper *et al*., 2023). TALEs bind to matching *EBEs* through a variable number of nearly identical, tandemly arranged 33–34 amino acid long peptide modules commonly referred to as repeats, with each TALE‐repeat aligning with one nucleotide of a matching *EBE*. Repeat variable diresidues (RVDs) located at positions 12 and 13 of each TALE repeat determine the base preference of the repeat. These base preferences have been decoded for all possible RVDs, and these RVD‐nucleotide correlations, commonly referred to as the TALE code, now allow *in silico* prediction of DNA target sites for any given TALE protein (Teper *et al*., 2023).

The mechanistic basis of how TALEs recruit the host's RNA polymerase II (pol II) complex has also been elucidated in recent studies. For the initiation of transcription, pol II assembles with several general transcription factors, including TFIIA, at the promoter DNA to form the pre‐initiation complex (PIC; Girbig *et al*., 2022). TALEs bind to this pol II PIC with their transcription factor binding domain (TFB), which is located C‐terminal to the DNA binding domain of the TALE (Yuan *et al*., 2016). The TFB domain interacts with TFIIAγ, which is a subunit of the general transcription factor TFIIA. Examination of the three‐dimensional architecture of the *Saccharomyces cerevisiae* pol II PIC reveals that TFIIA is located close to the upstream promoter DNA (Plaschka *et al*., 2016), consistent with a model in which the TALE protein physically bridges the distance between a given *EBE* and the pol II PIC. The model in which TALEs physically link pol II PICs and *EBEs* is consistent with the observation that TALE‐induced transcripts are generally initiated ~50 nucleotides downstream of a given *EBE* (Antony *et al*., 2010; Hummel *et al*., 2012; Römer *et al*., 2009a,b; Strauß *et al*., 2012; Tian *et al*., 2014; Tran *et al*., 2018; Wang *et al*., 2015).

Virulence of the CBC‐causing pathogen *Xcc* largely depends on the TALE protein PthA4. PthA4 binds to a compatible *EBE* upstream of the *Citrus sinensis lateral organ boundaries 1* (*CsLOB1*) gene and induces transcription of the downstream host *S* gene, which encodes a LOB‐transcription factor (Hu *et al*., 2014; Li *et al*., 2014). How CsLOB1 expression benefits the bacterial pathogen remains unclear. However, since PthA4‐dependent activation of the virulence‐promoting *CsLOB1* gene depends on PthA4‐compatible *EBEs* upstream of *CsLOB1*, CRISPR‐based mutagenesis of the *PthA4‐EBE* is expected to reduce the virulence of PthA4‐dependent *Xcc* strains. Recent studies using CRISPR mutagenesis to mutate PthA4‐binding *EBEs* in the *CsLOB1* promoter have indeed shown corresponding citrus plants to have increased resistance to *Xcc* (Huang *et al*., 2021, 2022; Jia *et al*., 2016, 2017, 2022a,b; Peng *et al*., 2017). Given that optimal *Xcc* growth in citrus depends on TALE‐mediated activation of *CsLOB1*, one would expect strong evolutionary pressure on *Xcc* to maintain the ability to activate *CsLOB1* through TALEs. Indeed, recent studies have shown that *Xcc* strains harbouring PthA4 derivatives that are no longer able to activate *CsLOB1* due to mutations in their DNA‐binding domains can alter their DNA‐binding domains after prolonged incubation in host plants to regain the ability to activate *CsLOB1* (Teper and Wang, 2021). Given the high evolvability of the TALE DNA‐binding domain, it is therefore likely that CRISPR‐induced mutations in the *PthA4‐EBEs* of the *CsLOB1* promoter would exert evolutionary pressure on *Xcc*. This would likely lead to the selection of *in planta*‐evolved TALEs (eTALEs) with altered DNA‐binding specificity capable of transcriptionally activating CRISPR‐induced *CsLOB1* mutant alleles. In conclusion, CRISPR‐induced mutagenesis of *PthA4‐EBEs* in the *CsLOB1* promoter is unlikely to confer durable resistance to *Xcc*.

While CRISPR‐induced *EBE* mutations suppress the virulence‐promoting function of TALEs, TALE‐triggered immune responses offer an alternative way to suppress *in planta* growth of TALE‐carrying xanthomonads. Molecular analysis of dominantly inherited TALE‐triggered plant‐immune reactions revealed two functionally distinct classes of *R* genes: (i) constitutively expressed *R* genes encoding nucleotide‐binding leucine rich repeat (NLR) proteins and (ii) transcriptionally regulated executor *R* genes. Tomato (*Lycopersicum esculentum*) Bs4 and rice (*Oryza sativa*) Xa1/Xo1 are representatives of such TALE‐recognizing NLRs, which achieve specific recognition of TALEs presumably through direct interaction (Ji *et al*., 2020; Read *et al*., 2020a,b; Schornack *et al*., 2004; Triplett *et al*., 2016). While the molecular basis of NLR‐dependent TALE recognition remains to be elucidated, the molecular processes by which executor type *R* genes recognize microbial TALE proteins are well understood. The *EBEs* in the promoters of the executor *R* genes act as TALE traps that misdirect the virulence activity of the TALEs to induce the transcription of resistance‐mediating executor transcripts that induce cell death and stop the spread of the biotrophic parasite. Accordingly, such executor *R* genes are also often referred to as promoter traps. Previous studies have shown that the recognition capacity of executor *R* genes can be expanded by incorporating multiple back‐to‐back *EBEs* into the *R* gene promoter, resulting in executor *R* genes that can mediate the recognition of multiple TALEs (Hummel *et al*., 2012; Römer *et al*., 2009a; Zeng *et al*., 2015). These may be *EBEs* that recognize TALEs from different strains of *Xanthomonas*, resulting in broad‐spectrum resistance, or *EBEs* that detect several or all the numerous TALEs from a particular strain of *Xanthomonas*. The latter configuration, in which multiple TALEs of a *Xanthomonas* strain each independently activate the *R* promoter, should confer durable resistance because all *TALE* genes encoding promoter‐activating proteins must mutate simultaneously to evade recognition by this enhanced executor *R* gene. Given that recent studies have shown the high evolutionary capacity of TALEs under conditions of high evolutionary selection pressure, such designed executor *R* genes that are preceded with an arsenal of TALE‐trapping *EBEs* will possibly confer durable resistance to *Xcc* (Teper and Wang, 2021).

Previously, we engineered an enhanced executor *R* gene to confer broad spectrum and durable resistance to CBC (Shantharaj *et al*., 2017). To establish this promoter trap, we used a repertoire of 14 different TALEs from citrus‐infecting xanthomonads, derived matching *EBEs* using the TALE code, and inserted a corresponding *EBE* array into the promoter of the pepper executor *R* gene *Bs3*. This *R* gene promoter controls the expression of *avrGf1*, which encodes a *Xanthomonas* effector protein that triggers the hypersensitive response (HR) in grapefruit (*Citrus paradisi*; Rybak *et al*., 2009). Using transient, Agrobacterium‐mediated expression in citrus, we confirmed that this designed executor *R* gene, termed *ProBs3*_*14EBE*_ *:avrGf1*, indeed mediated recognition of a broad spectrum of *Xcc* strains. However, in these previous studies, we were unable to identify a stable transgenic line containing *ProBs3*_*14EBE*_ *:avrGf1*.

We present here the establishment and molecular characterization of a stable‐transgenic grapefruit line containing a derivative of *ProBs3*_*14EBE*_ *:avrGf1*, which we have termed *Xcc‐TALE‐trap*. While the TALE‐sensing promoter is identical in both executor *R* genes, the *Xcc‐TALE‐trap* now contains *avrGf2* instead of *avrGf1*, a *Xanthomonas* effector that, based on previous studies, induces a stronger HR in grapefruit than *avrGf1* (Gochez *et al*., 2015) and is therefore potentially a better executor of cell death.

To gain insight into the recognition specificity of tandemly arranged *EBEs* in our designed promoter trap, we performed rapid amplification of cDNA ends (RACE), an approach that allows us to determine which *EBEs* have been chosen for transcription of a given TALE‐induced transcript. These findings showed that in addition to their designated high‐affinity *EBEs*, TALEs often bind to sequence‐related *EBEs*. We also studied the *Xcc‐TALE‐trap* in conjunction with *in planta*‐evolved derivatives of the *Xcc* TALE protein PthA4. These studies suggest that the *Xcc‐TALE‐trap* recognizes not only current but also newly evolved eTALE proteins. Finally, we show that the *Xcc‐TALE‐trap* confers *Xcc* resistance not only in laboratory assays but also in more agronomically relevant field studies.

## Results

### Construction of a synthetic plant *R* gene confers citrus canker resistance

**Figure 1:**
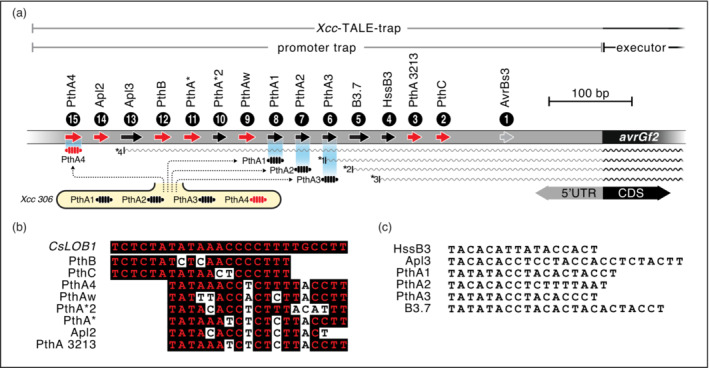
A promoter trap designed to perceive various *X. citri* TALE proteins controls transcription of *Xanthomonas avrGf2*, encoding an effector protein that triggers cell death in grapefruit. (a) Each of the four different *Xcc306* TALEs can independently activate the *Xcc‐TALE‐trap*. White framed arrows show *EBEs* along with TALEs which were used to derive these *EBEs*. To make it easier to address individual *EBEs*, they are also identified by a simple one‐digit code (white font in black circle). Red arrows represent *EBEs* of CsLOB1‐targeting TALEs. Black arrows indicate TALE *EBEs* whose host target gene is unknown. The yellow oval represents strain *Xcc306*, which delivers the four TALE proteins shown. Each *Xcc306* TALE protein is expected to bind to a complementary high‐affinity *EBE* and induce a transcript ~50 base pairs downstream encoding the cell death inducing AvrGf2 protein (wavy line). The binding of *Xcc306* TALEs to complementary *EBEs* is indicated by a blue background colour. The start sites of TALE‐induced transcripts, located ~50 nucleotides downstream of the respective TALE *EBEs*, are indicated by numbered asterisks. (b) Nucleotide sequences of *EBEs* designed to capture matching, CsLOB1‐targeting TALE proteins. The *CsLOB1* promoter (uppermost sequence) and *EBEs* along with matching TALEs have been incorporated in the promoter trap. (c) Nucleotide sequences of TALE *EBEs* in the promoter trap whose host target genes are unknown.

To generate a synthetic plant *R* gene that triggers HR upon the perception of TALEs from *Xcc*, here referred to as the *Xcc‐TALE‐trap*, we placed the *Xanthomonas avrGf2* gene, which triggers HR in citrus, downstream of 14 tandem‐arranged *EBEs* that were designed using the TALE‐code to have high affinity to known *X. citri* TALEs (Figure 1). Eight of the 14 *EBEs* were designed to mediate the recognition of different *Xcc* TALEs, each of which can autonomously activate the disease‐promoting *CsLOB1* gene (Figure 1; red arrows). The remaining six *EBEs* were designed to trap distinct *Xcc* TALEs for which the plant target genes are currently unknown but for which matching *EBEs* can be predicted by the TALE code (black arrows). In addition to these 14 *EBEs* matching to *Xcc* TALEs, we incorporated an *EBE* matching to AvrBs3 (grey arrow), a well‐studied TALE protein from the pepper pathogen *Xanthomonas euvesicatoria* (*Xeu*; Bonas *et al*., 1989). Generally, *Xcc* strains contain several TALEs (Teper *et al*., 2023), with one or more TALEs likely being compatible with *EBEs* in the *Xcc‐TALE‐trap* promoter and each individually capable of activating transcription of the HR‐inducing executor gene *avrGf2*. For example, strain *Xcc306* contains next to the *CsLOB1*‐activating TALE protein PthA4 three additional trap‐activating TALEs (PthA1, PthA2, and PthA3). Given that in our *Xcc‐TALE‐trap* promoter we incorporated for each of these four *Xcc306* TALEs a corresponding TALE‐code predicted *EBE*, each of these four TALEs is expected to trigger the trap independently (Figure 1). Since mutations in all four *Xcc306 TALEs* would be required to evade recognition by this *Xcc‐TALE‐trap*, it seems plausible that the *Xcc‐TALE‐trap* will provide durable resistance to *Xcc306* and presumably other *Xcc* strains containing more than one *Xcc‐TALE‐trap* compatible TALE. Seeing as the *Xcc‐TALE‐trap* contains TALE code predicted *EBEs* with high affinity for numerous currently known *Xcc* TALEs, this trap is also expected to provide broad‐spectrum *Xcc* resistance.

### The *Xcc‐TALE‐trap* inhibits development of *Xcc*‐related disease phenotypes

The *Xanthomonas* effector protein AvrGf2, which we integrated as a TALE‐inducible executor gene in the *Xcc‐TALE‐trap*, is known to trigger HR in citrus (Gochez *et al*., 2017). Accordingly, it was important to show that in the absence of the pathogen, there is no executor expression and that AvrGf2‐triggered HR is strictly TALE dependent. To test stringent transcriptional regulation of *avrGf2*, we inoculated *Agrobacterium tumefaciens* containing the *Xcc‐TALE‐trap* T‐DNA with or without *Xcc306* into grapefruit leaves. In these transient assays, the *Xcc‐TALE‐trap* induced HR only when inoculated together with *Xcc306*, and not when co‐inoculated with *XccΔ4* (Figure S1), an *Xcc306* derivative from which all four *TALE* genes were removed by mutagenesis (Hu *et al*., 2014). This observation suggests that the *Xcc‐TALE‐trap* triggers HR in a strictly TALE‐dependent fashion and therefore this T‐DNA construct seemed suitable for application in stably transformed citrus plants.

**Figure 2:**
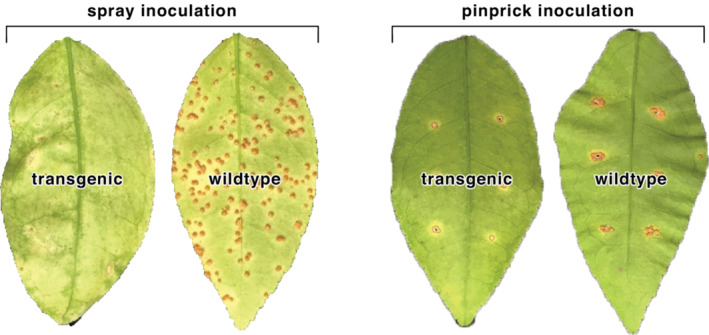
Transgenic grapefruit containing a promoter trap driving AvrGf2 expression show resistance to strain *Xcc306* when being inoculated by either pinprick inoculation or spray inoculation. Leaves of the transgenic line and wild‐type Duncan grapefruit were inoculated with *Xcc306* (5 × 10^8^ cfu/mL) containing the TALE proteins PthA1, PthA2, PthA3, and PthA4. Pictures were taken at 12 days post‐inoculation (dpi).

We initiated the transformation of Duncan grapefruit plants and identified within five putative transgenic lines one plant that indeed contains the *Xcc‐TALE‐trap* as a stable transgene (Figure S2). To test for citrus canker resistance, we inoculated this transgenic line and a corresponding Duncan grapefruit wild‐type control plant with *Xcc306*, an *Xcc* strain that is expected to activate the *Xcc‐TALE‐trap* with four distinct TALEs: PthA1, PthA2, PthA3, and the *CsLOB1*‐activating PthA4 protein (Figure 1). When spray inoculated, only wild‐type (WT) Duncan plants, but not the derived transgenic line, showed typical citrus canker pustules, a disease‐associated infection phenotype characteristic of susceptible plant genotypes (Figure 2). Similarly, infection of *Xcc306* via pinprick inoculation triggered canker‐like lesions only on leaves of Duncan WT plants but not on leaves of the derived transgenic line carrying the *Xcc‐TALE‐trap*. In summary, transgenic lines containing the *Xcc‐TALE‐trap* showed no disease phenotypes.

### The *Xcc‐TALE‐trap* inhibits *in planta* growth of *Xcc306*

**Figure 3:**
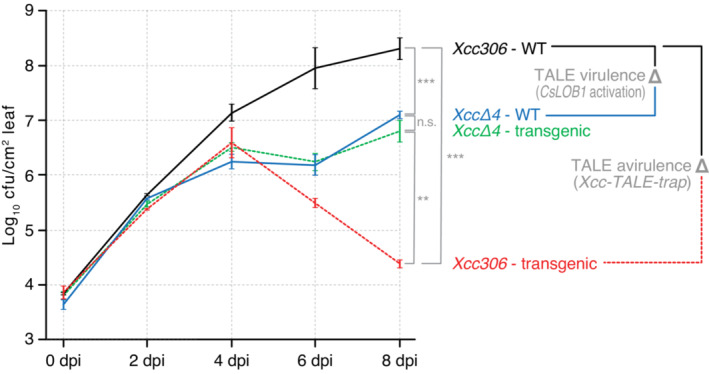
*In planta* growth of *Xcc* is inhibited in transgenic Duncan grapefruit plants containing the *Xcc‐TALE‐trap*. Bacterial suspensions were adjusted to 10^5^ cfu/mL prior to blunt‐end syringe infiltration of wild‐type (WT) and transgenic Duncan grapefruit leaves (transgenic). Leaf discs of inoculated tissue were assayed for bacterial populations of depicted *Xcc* strains at the depicted days post‐infiltration (dpi). Values are presented in a line graph with the mean value of three biological replicates (*n* = 3) and the error bars displaying the calculated standard deviation. Statistical significance on day 8 was determined between the indicated plant and bacterial genotype combinations using a Student's *t*‐test, ***P < 0.001, **P < 0.01, n.s.*P* > 0.05, no significance. Differences of *in planta* growth observed for the two studied *Xcc* strains are caused either by differences TALE‐dependent virulence activity (*Xcc306* vs. *XccΔ4* in WT host) or TALE‐dependent avirulence activity (*Xcc306* in WT vs. *Xcc306* in transgenic).

Given that disease phenotypes of *Xcc* were suppressed in the transgenic line, we wondered if the *Xcc‐TALE‐trap* would also inhibit *in planta* growth of *Xcc306*. To clarify if *in planta* growth of *Xcc306* differs in the context of leaves from Duncan WT and leaves from the transgenic line, we inoculated both plant genotypes and quantified bacterial multiplication over a period of eight days. This time course analysis showed that until four days post inoculation (dpi), *in planta* growth of *Xcc306* was almost indistinguishable in Duncan WT and the transgenic line (Figure 3). Yet, at six and eight dpi, the number of colony‐forming units that could be recovered from leaves declined in the context of the transgenic line and the inoculated leaf tissue generally showed HR at a later timepoint. By contrast, multiplication of strain *Xcc306* continued at six and eight dpi in Duncan WT plants. Notably, the number of colony‐forming units recovered at eight dpi from Duncan WT and the transgenic line differed by almost four logs, demonstrating the highly efficient bacterial resistance conferred by the *Xcc‐TALE‐trap*.

Next, we tested whether the reduced *in planta* growth of strain *Xcc306* in the context of the transgenic line is TALE dependent. To clarify TALE dependence, we used strain *Xcc306* and *XccΔ4*, an isogenic *Xcc306* derivative lacking *TALE* genes (Hu *et al*., 2014). Inoculation studies of Duncan WT and the transgenic line with *XccΔ4* revealed that this TALE‐depleted *Xcc* strain multiplied in both plant genotypes to almost identical levels (Figure 3). This observation suggests that the reduction of *in planta* growth of *Xcc306* in the context of the transgenic line is indeed TALE‐dependent. We also noted that in Duncan WT plants, growth of *XccΔ4* was reduced relative to growth of *Xcc306*. The reduced *in planta* growth of the TALE‐depleted mutant *XccΔ4* is in accordance with previous studies where PthA4 and other *Xcc* TALEs were shown to promote *in planta* growth of *Xcc* (Hu *et al*., 2014).

**Figure 4:**
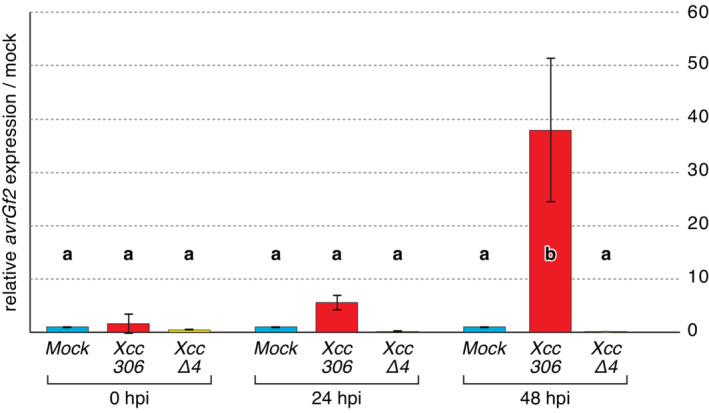
Analysis of the transgenic grapefruit line shows that the executor gene *avrGf2* is transcriptionally activated by the *Xcc306* wild‐type strain but not by a derived mutant strain (*XccΔ4*) lacking *pthA14*. *avrGf2* transcript levels were quantified by quantitative reverse‐transcription real‐time PCR. *avrGf2* expression was quantified at 0, 24, and 48 hours post‐inoculation (hpi) with either *Xcc306* (5 × 10^8^ cfu/ml), its mutant derivative *XccΔ4* (5 × 10^8^ cfu/mL) or inoculation medium (mock). *avrGf2* transcript levels were normalized to *Ef1α* expression and relative to mock treatment. Data are presented in a bar graph with the mean value of two biological replicates (*n* = 2) and error bars displaying the calculated standard deviation. Statistically significant groups (*P* < 0.01) are indicated with lower case letters calculated according to one‐way ANOVA followed by a Tukey HSD *post hoc* test.

Based on the design of the *Xcc‐TALE‐trap*, the observed HR should generally correlate with transcriptional activation of the *avrGf2* executor transgene by *Xcc*‐delivered TALE proteins (Figure 1). To test TALE‐dependent transcriptional activation of the *avrGf2* transgene, we inoculated leaves of the transgenic citrus line with *Xcc306*, its *TALE* gene‐depleted derivative *XccΔ4* or with inoculation medium. Next, we harvested inoculated leaf tissue at 0, 24, and 48 hours post inoculation (hpi) and determined *avrGf2* transcript levels by quantitative reverse‐transcription PCR (qRT‐PCR). qRT‐PCR studies showed elevated *avrGf2* transcript levels in the transgenic line at 24 and 48 hpi with  *Xcc306* (Figure 4). By contrast, inoculation of *XccΔ4* or inoculation medium did not induce elevated *avrGf2* transcript levels in the transgenic line. In summary, these studies show that the *Xcc‐TALE‐trap* is transcriptionally activated by *Xcc306* TALE proteins.

To determine if in the transgenic line AvrGf2‐triggered HR interferes with PthA4‐dependent activation of *CsLOB1*, transgenic and non‐transgenic grapefruit were inoculated with *Xcc306* or the *TALE* gene‐deleted strain *XccΔ4*. Quantification of transcripts at 48 hpi revealed that *Xcc306* but not *XccΔ4* induced elevated *CsLOB1* levels in both, transgenic and non‐transgenic grapefruit (Figure S3). However, the increase in *CsLOB1* was lower in the transgenic grapefruit than in the non‐transgenic grapefruit, probably as a result of initiation of the HR in the transgenic plant, which is likely to have an inhibitory effect on all biochemical processes, including transcription. In conclusion, we show that both the *Xcc‐TALE‐trap* and *CsLOB1* are transcriptionally activated by *Xcc306* TALE proteins.

### The *Xcc‐TALE‐trap* is triggered by *Xcc*‐ but not by *Xoo*‐TALE proteins

To clarify whether each of the four *Xcc306* TALE proteins can trigger the *Xcc‐TALE‐trap*, we studied the TALE‐depleted *Xcc306* derivative *XccΔ4* and corresponding transconjugants containing the *TALE* genes *pthA1*, *pthA2*, *pthA3*, *pthA4*, or *avrBs3*. As anticipated, inoculation of the *TALE*‐depleted strain *XccΔ4* did not trigger HR in Duncan WT leaves nor in leaves of the transgenic line, demonstrating that *Xcc‐TALE‐trap* activation is *TALE*‐dependent (Figure S4). By contrast, each of the *XccΔ4* transconjugants carrying the individual *TALE* genes *pthA1*, *pthA2*, *pthA3*, *pthA4*, or *avrBs3*, triggered HR in the transgenic line containing the *Xcc‐TALE‐trap* but not in leaves of Duncan WT plants. These findings demonstrate that each of the *Xcc306* TALEs as well as the *Xeu* TALE AvrBs3 is sensed by and activates transcription of the *Xcc‐TALE‐trap*. Given that all tested *Xcc306* TALEs and AvrBs3 were recognized by the *Xcc‐TALE‐trap*, we wondered if other TALEs, for which matching *EBEs* were not incorporated in the trap promoter, would also activate the *Xcc‐TALE‐trap*. To study recognition specificity of the *Xcc‐TALE‐trap*, we used *XccΔ4* transconjugants carrying the *TALE* genes *pthXo1*, *pthXo6*, or *avrXa7* that were originally identified in the rice pathogen *X. oryzae* pv. *oryzae* (*Xoo*). Notably, these three *Xoo* TALEs differ in their DNA binding preference from *Xcc* TALEs and therefore are not expected to trigger the *Xcc‐TALE‐trap*. Indeed, inoculation of the *XccΔ4* transconjugants carrying the *TALE* genes *pthXo1*, *pthXo6*, or *avrXa7* did not trigger HR in the transgenic line containing the *Xcc‐TALE‐trap* (Figure S5), suggesting that our promoter trap recognizes TALE proteins specifically from *Xcc*.

### The *Xcc‐TALE‐trap* mediates broad‐spectrum resistance to *Xcc* strains

**Figure 5:**
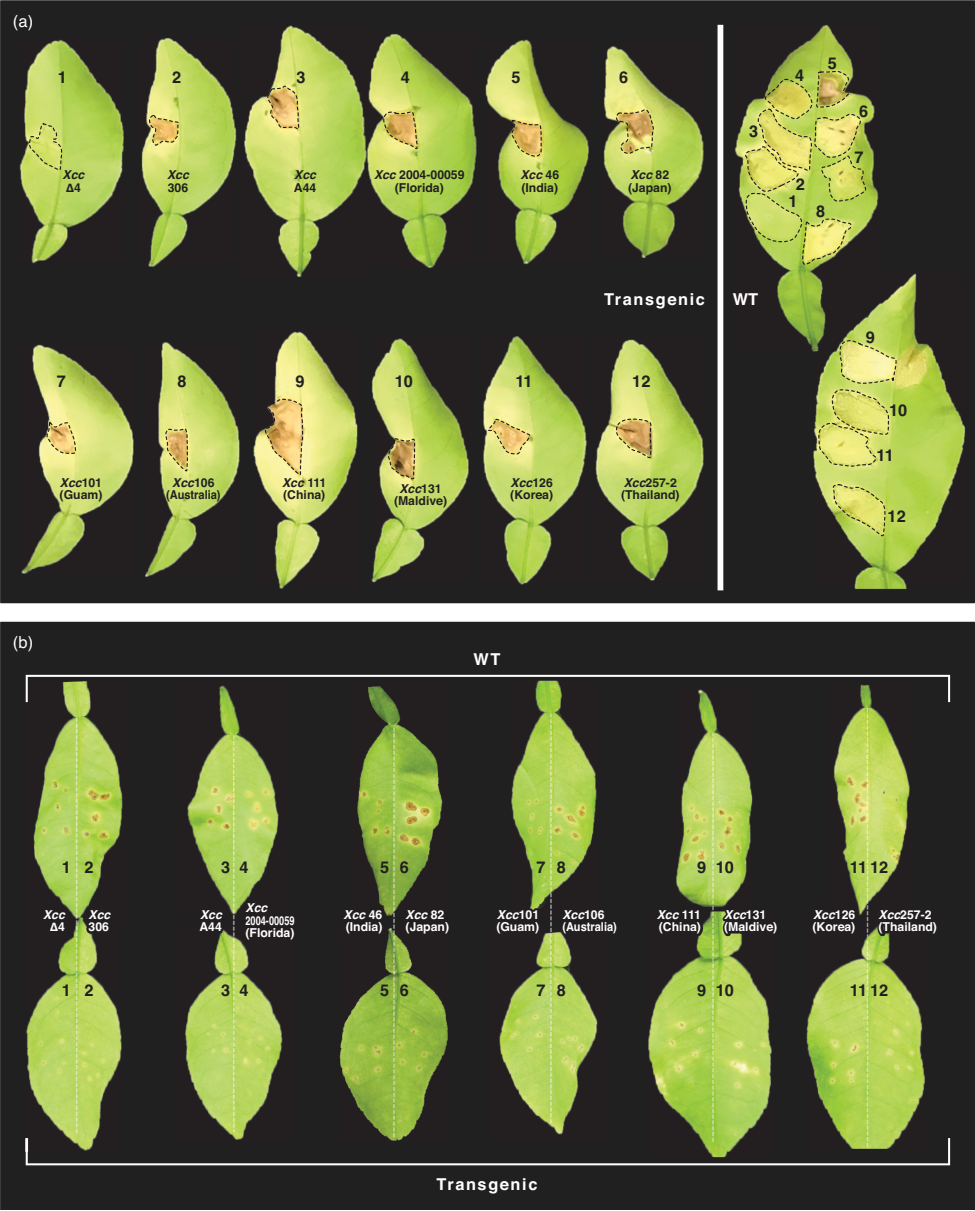
A transgenic Duncan line containing the *Xcc‐TALE‐trap* mediates recognition to a broad panel of *Xcc* strains. Leaves of either Duncan wild‐type (WT) or a transgenic line containing the *Xcc*‐*TALE‐trap* (transgenic) were infected with a panel of depicted *Xcc* strains (5 × 10^8^ cfu/mL) using infiltration (a) or pinprick inoculation (b). Pictures were taken at 4 (a) or 12 (b) dpi.

The *Xcc‐TALE‐trap* was engineered using a spectrum of 14 sequenced *Xcc* TALE proteins for which corresponding *EBEs* were deduced by the TALE code and integrated into the promoter trap (Figure 1). Given that characterized *Xcc* strains typically contain several TALEs, we speculated that the *Xcc‐TALE‐trap* would possibly mediate resistance to a broad range of *Xcc* strains. To test the recognition spectrum of the *Xcc‐TALE‐trap*, we studied a panel of 10 distinct *Xcc* strains that originate from countries across four different continents (South America, North America, Asia, and Oceania) and that likely cover at least some genetic diversity observed within *Xcc* (Table S1). While the *TALE* gene repertoire of these strains is unknown, Southern analysis using the *Xcc306 PthA4* gene as a probe showed that all strains in this collection contain *TALE* genes (Figure S6) and should therefore activate the *Xcc‐TALE‐trap*. In our infection‐based studies of the *Xcc* collection, we included the well‐characterized strain *Xcc306*, and the derived TALE‐depleted strain *XccΔ4* as positive and negative controls respectively. The panel of *Xcc* strains was inoculated into Duncan WT plants, as well as the transgenic line containing the *Xcc‐TALE‐trap*, using syringe infiltration as well as pinprick inoculation (Figure 5). When using syringe infiltration, all *Xcc* strains except *XccΔ4* triggered HR in the transgenic line, but not in Duncan WT plants. When using pinprick inoculation, the severity of disease‐associated pustules on Duncan WT showed some variation across the strains and was not evident in the transgenic line. Overall, our findings suggest that the *Xcc‐TALE‐trap* mediates broad‐spectrum resistance to CBC.

### 5′ RACE studies suggest that TALE proteins bind preferentially to TALE‐code predicted *EBEs*

**Figure 6:**
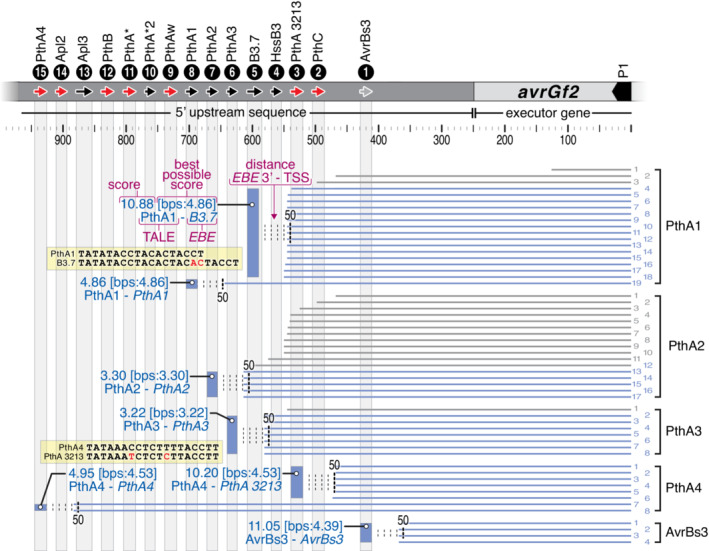
5′ RACE studies suggest that TALEs do not bind exclusively to the highest affinity target site of the *Xcc‐TALE‐trap*. The grey horizontal bar on top depicts the promoter trap consisting of the *avrGf2* executor gene (far right) and the 5′ upstream sequence with distinct tandem‐arranged *EBE*s (arrows). Each *EBE* is labelled with the TALE that was used to deduce this *EBE* and for simplicity with a number (white font on black background). Horizontal grey and blue lines with numbering on the far right (3′ end) represent 5′ RACE products with the variable 5′ end on the far left. Transcript‐inducing TALEs are indicated next to the square brackets on the far right. Vertical grey bars originating from the *EBE*s indicate the location of the *EBEs* relative to the 5′ transcript ends. Transcripts for which an *EBE* could be predicted ~50 nucleotides downstream of the 5′ transcript end by Target Finder (https://tale-nt.cac.cornell.edu/node/add/talef-off) are depicted in blue colour along with the corresponding predicted *EBE* shown as blue vertical boxes. The score of predicted TALE‐*EBE* combinations is given along with the best possible score (bps) for a given TALE in blue font next to the given *EBE*. Detailed representative descriptions on TALE‐*EBE* combinations are also given in purple‐font text on the uppermost TALE‐*EBE* combination. Yellow boxes show sequence alignments of TALE‐code‐predicted *EBEs* and chosen *EBEs* for PthA1 and PthA4. P1; anchor primer used in 5′ RACE studies. *Xcc* strains (5 × 10^8^ cfu/ml) were inoculated blunt‐end syringe infiltration; inoculated tissue for transcript studies was harvested at 48 hpi.

The promoter of the *Xcc‐TALE‐trap* contains 14 tandem‐arranged *EBEs* designed to capture *Xcc* TALEs with distinct DNA‐binding specificity (Figure 1). However, it remains unclear whether or not the different *Xcc*‐delivered TALEs in a native infection scenario exclusively or at least preferentially target the TALE code predicted *EBEs*. This raises the question of how to determine which of the tandem‐arranged *EBEs* in the *Xcc‐TALE‐trap* in fact captures a given TALE protein. Numerous studies have shown that the transcriptional start site (TSS) of TALE‐induced transcripts is generally ~50 nucleotides downstream of the targeted *EBE* (Antony *et al*., 2010; Kay *et al*., 2007; Römer *et al*., 2007, 2009a,b; Streubel *et al*., 2017). Accordingly, the TSS of a TALE‐induced transcript can be used to identify a corresponding TALE‐binding *EBE* in the promoter trap. To clarify which of the 14 tandem‐arranged *EBEs* in fact interacts with matching TALEs, we studied five TALEs for which TALE‐code predicted, perfect‐match *EBEs* (*pEBEs*) had been incorporated into the promoter trap: AvrBs3 a TALE from the tomato and pepper pathogen *Xeu*, as well as PthA1, PthA2, PthA3, and PthA4, four TALEs from the citrus‐infecting strain *Xcc306*. To study TALE‐*EBEs* interactions for the *Xcc‐TALE‐trap*, *XccΔ4* transconjugants containing either *avrBs3*, *pthA1*, *pthA2*, *pthA3*, or *pthA4* were inoculated into the transgenic Duncan grapefruit line. Two dpi, RNA was extracted from inoculated leaf tissues and rapid amplification of cDNA ends (RACE) was carried out. PCR‐amplified *avrGf2* transcripts were cloned and sequenced to determine TSSs for each of the five *Xcc*‐delivered TALEs. Inspection of the executor transcript 5′ ends induced by the five studied TALEs uncovered for all TALEs corresponding transcripts starting sites ~50 nucleotides downstream of their *pEBEs* (Figure 6; Figure S7). For two of the five TALEs that were studied, AvrBs3 and PthA3, the majority of TSSs were ~50 nucleotides downstream of their designated *pEBEs*, which corroborates TALE‐code predictions. Since the TSSs for most of the PthA1‐, PthA2‐, and PthA4‐induced transcripts were not ~50 nucleotides downstream of their designated *pEBE*, we assumed that some TSSs could be explained by sequences in the promoter that are highly sequence related to the designated *pEBEs*. To test this hypothesis, we scanned the promoter sequences located ~50 bp upstream of the observed TSSs using the TARGET FINDER algorithm (Doyle *et al*., 2012) to identify ‘second best’ *EBEs* (*sEBEs*) for the given TALEs. Indeed, TARGET FINDER uncovered for both PthA1 and PthA4 such *sEBEs* that are in accordance with the observed TSSs. For example, 15 of the 19 PthA1‐induced transcripts start ~50 bp downstream of the B3.7‐*EBE* (*EBE5*) which differs from the *PthA1‐pEBE* (*EBE8*) in only two out of 18 nucleotides. Similarly, the TSSs of six of the eight PthA4‐induced transcripts starts ~50 bp downstream of the *PthA3213‐EBE* (*EBE3*), which differs from the *PthA4‐pEBE* (*EBE15*) in only two out of 18 nucleotides. While for four out of five TALEs, the majority of observed TSSs could be explained by the TALE code, the situation was different for PthA2. With PthA2, only five of 17 TSSs were ~50 nucleotides downstream of its *pEBE* (*EBE7*), and the remaining 12 TSSs could not be explained by TALE‐code predictions. In summary, most observed TALE‐induced executor transcripts can be explained by interaction of the TALEs with code predicted *pEBEs* or sequence‐related *sEBEs*.

### Newly evolved *Xcc* TALEs transcriptionally activate the *Xcc‐TALE‐trap*

**Figure 7:**
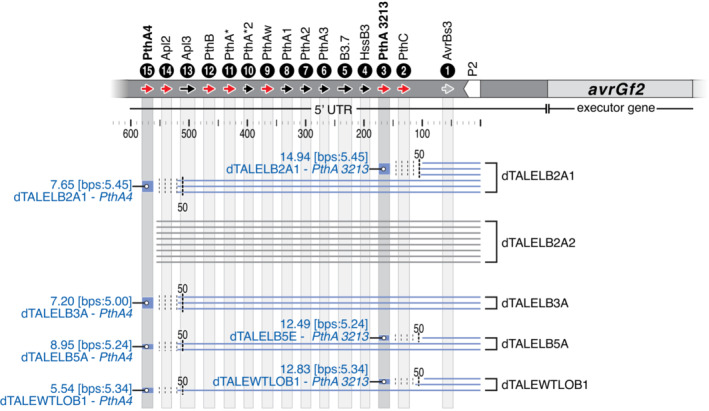
5′ RACE studies suggest that the *Xcc‐TALE‐trap* is capable of detecting evolved TALE proteins. For explanations of the graphics, please refer to Figure 6.

The *Xcc‐TALE‐trap* contains *pEBEs* corresponding to eight *Xcc* TALEs that are known to transcriptionally activate the disease‐promoting *CsLOB1* gene, including PthA4 from strain *Xcc306* (Figure 1). Recent studies uncovered that PthA4 derivatives with mutated DNA binding domains that are incapable of activating *CsLOB1*, rapidly evolve to regain the capability to transcriptionally activate *CsLOB1* (Teper and Wang, 2021). While the DNA‐binding domain of these PthA4‐derived eTALEs and the wild‐type PthA4 protein differ, the eTALEs indeed share high similarity in the RVD composition of their DNA binding repeat arrays with PthA4 (Figure S8). To clarify if the PthA4‐derived eTALEs dTALELB2A1, dTALELB2A2, dTALELB3A, dTALELB5A, and their progenitor dTALEWTLOB1 would be captured by *EBEs* in the *Xcc‐TALE‐trap* that were designed to trap wild‐type PthA4 protein (*EBE15*), we delivered the TALEs into transgenic Duncan grapefruit via *Xcc* and carried out RACE as previously described. Analysis of TSSs showed that three out of four eTALEs (dTALELB2A1, dTALELB3A, and dTALELB5A) and their progenitor dTALEWTLOB1 bound to the PthA4 *pEBE* (*EBE15*) of the *Xcc‐TALE‐trap*. dTALELB2A1, dTALELB5A, and dTALEWTLOB1 induced additional transcripts that indicated interaction of these PthA4 derivatives with the *PthA3213‐EBE* (*EBE3*) (Figure 7), as was the case for the wild‐type PthA4 protein (Figure 6). By contrast, the TSSs of dTALELB2A2‐initiated transcripts could not be associated with Target Finder‐predicted upstream *EBEs*. In summary, three out of four of the PthA4‐derived eTALEs (dTALELB2A1, dTALELB3A and dTALELB5A) showed interactions with *EBEs* of the *Xcc‐TALE‐trap* that were similar to the progenitor TALE PthA4.

### The *Xcc‐TALE‐trap* mediates resistance to citrus canker in a field study

While our laboratory infection studies suggested that the *Xcc‐TALE‐trap* would confer resistance to *Xcc* (Figures 2, 3, and 5), such experiments conducted under laboratory conditions lack the complexity of the situation observed in the field. Therefore, we initiated studies to determine whether the transgenic line outperformed Duncan grapefruit, the progenitor of the transgenic line, under field conditions. Following planting in March 2019, a visual inspection 3 months later in June 2019 revealed no canker symptoms in the transgenic line and very few in the Duncan grapefruit (Table S2). However, inspections after six and nine months in September and December of 2019 revealed moderate to severe disease symptoms on Duncan grapefruit, but not on any of the derived transgenic lines carrying the *Xcc‐TALE‐trap* (Table S2). Therefore, our field studies show that transgenic lines carrying the *Xcc‐TALE‐trap* provide CBC resistance not only in controlled laboratory infection assays but also under agronomically relevant field conditions.

## Discussion

### Towards cisgenic canker‐resistant citrus plants

We established and characterized a transgenic citrus plant containing the *Xcc‐TALE‐trap*, a synthetic executor type *R* gene designed to trigger HR in host cells upon perception of *Xcc* TALE proteins. The transgenic citrus line established in this study is based on previously published work in which a promoter trap for *Xcc* TALE proteins was analysed by transient expression rather than in stable transgenic lines (Shantharaj *et al*., 2017). In this work, the same engineered promoter was used, but a different cell death inducing effector, AvrGf2, was used instead of the previously used AvrGf1 protein. AvrGf1 and AvrGf2 share 45% sequence similarity and both effectors trigger HR in most citrus species, except key lime (*C. aurantifolia*; Gochez *et al*., 2015). Quantitative studies revealed that AvrGf2 triggers a stronger HR and mediates a stronger inhibition of *in planta* growth of *Xcc* as AvrGf1 (Gochez *et al*., 2015), possibly suggesting that AvrGf2 is the more potent executor protein. Agrobacterium‐mediated transient delivery of *ProBs3*_*14EBE*_ *:avrGf1* and the *Xcc‐TALE‐trap* showed that both constructs execute HR in a strictly TALE‐dependent fashion (Figure S1) (Shantharaj *et al*., 2017), suggesting that the AvrGf1‐ and AvrGf2‐based executor type *R* genes are both equally suitable for application in stable transgenic lines. However, we failed to observe transgenic citrus lines for *ProBs3*_*14EBE*_ *:avrGf1* (Shantharaj *et al*., 2017) while we eventually identified one line containing the AvrGf2‐based *Xcc‐TALE‐trap* (Figure S2). The low overall transformation success rate might reflect the fact that the transformation of citrus is still challenging (Conti *et al*., 2021). However, recent studies have shown that transgenic application of the rice executor gene *Xa23* leads reduced bacterial growth, probably due to upstream promoters at the specific transgene integration site (Ji *et al*., 2022). Therefore, the low number of citrus transformants may also be related to the cellular toxicity of executor genes, which are likely to eliminate all transgenic lines with leaky executor transgene expression.

Since no executor genes have been identified in citrus plants, we used the bacterial effector gene *avrGf2* as a TALE‐inducible executor gene in our transgenic citrus lines. While the integration of foreign DNA into plant genomes suffers from low public acceptance (Sharma *et al*., 2022), it is important to note that transcription of this bacterial transgene was found *in planta* exclusively after infection with TALE gene carrying *Xcc* strains (Figure 4). Given the superb resistance of our transgenic line to *Xcc* infection, it is foreseeable that in plantations containing only transgenic trees, the bacterial *avrGf2* gene would not actually be transcribed, which should potentially eliminate or reduce public concerns. Since the rejection of gene editing (GE) approaches in plants by a large part of the public is probably the consequence of a lack of knowledge about the causal relationships between RNA, DNA, and protein, the concept of TALE‐activated immunity opens up an opportunity to improve the public's understanding and appreciation of the nuances of GE technology.

If executor genes were available from citrus, they could be used to generate cisgenic, rather than less accepted transgenic, executor type *R* genes. While a citrus executor has not been cloned as of yet, recent studies resulted in the identification of PthA4^AT^, a derivative of the *Xcc* TALE protein PthA4 that triggers HR in citrus (Roeschlin *et al*., 2019). Notably, PthA4^AT^ derivatives, which due to mutations in their nuclear localization signals do not translocate to the host nucleus, do not trigger HR, ultimately suggesting that PthA4^AT^ transcriptionally activates a to‐be‐identified citrus executor gene. In summary, these observations suggest that the citrus genome contains executor genes which, once cloned, could serve as modules for the construction of cisgenic executor genes for the transformation of citrus.

Mechanistic insights into NLR proteins point to another source of executor genes that have not yet been exploited for executor *R* gene construction. Upon recognition of microbial effectors, NLR proteins change their conformation from a quiescent to an activated state, which in turn executes immune programmes that typically result in HR. Mutational studies have led to the identification of conserved amino acids that can be manipulated to generate autoactive NLR variants that induce immune responses upon translation in the absence of an NLR‐activating effector (Maruta *et al*., 2022). Genes encoding autoactive NLRs are functionally equivalent to the executor genes and could be used for the construction of TALE‐inducible *R* genes. Notably, the recently published citrus genomes (Wu *et al*., 2022) provide easy access to numerous genes encoding NLRs that could be used as starting materials to deduce autoactive citrus NLRs.

We also anticipate that research on cell death in the context of developmental biology will lead to the discovery of previously unknown executors, particularly in plant model species (Nowack *et al*., 2022). Subsequent identification of orthologous executor genes from crop species will allow us to establish TALE‐inducible executor type *R* genes through a cisgenic rather than transgenic approach in citrus and other crop species that hopefully will no longer raise public concern.

### Analysis of executor transcript TSSs provides insights into TALE‐targeted *EBEs* in the *Xcc‐TALE‐trap*

The promoter of our *Xcc‐TALE‐trap* is equipped with an arsenal of 14 tandem‐arranged *EBEs* that were designed to have high affinity to one of 14 distinct *Xcc*‐TALEs (Figure 1). Similarly, five and six tandem‐arranged *EBEs* were previously integrated into the promoters of the rice executor *R* genes *Xa10* and *Xa27*, respectively, in order to extend their recognition capacity for TALE proteins (Hummel *et al*., 2012; Zeng *et al*., 2015). A unique feature of the *Xcc‐TALE‐trap* is its *EBE* array and how it addresses functionally related TALEs that target identical or overlapping sequences in the host plant as, for example, PthA4, PthAw, PthA*2, PthA*, Apl2, and PthA 3213 (Figure 1b). Notably, for each of these functionally related TALEs, the *EBE* array of the *Xcc‐TALE‐trap* contains a TALE code‐predicted perfect match *EBE*, rather than a ‘broad spectrum’ *EBE* that is likely less efficient in detecting all of these functionally related TALEs (Table S3). We reasoned that this arrangement would improve the chances that TALEs with related but distinct DNA‐binding preferences would be matched with high‐affinity *EBE*s, potentially improving the sensitivity of the *Xcc‐TALE‐trap*. To clarify if *Xcc* TALEs would bind exclusively to their designated high‐affinity *pEBE* or also to sequence‐related *EBEs* with presumably lower affinity, we studied five distinct TALEs for which matching *pEBEs* were incorporated in the promoter of the *Xcc‐TALE‐trap*. 5′ RACE studies indicated that some but not all executor transcripts are initiated by TALEs at their designated *pEBE* (Figure 6). For example, two out of eight PthA4‐induced executor transcripts were initiated ~50 nucleotides downstream of the destined *PthA4‐EBE* (*EBE3*), while five out of eight executor transcripts were initiated ~50 nucleotides downstream of the *PthA3213‐EBE* (*EBE15*). Given that these two *EBEs* differ in only two of 19 nucleotides, it seems plausible that PthA4 can interact with both *EBEs*. Yet, TALE‐code based *in silico* predictions suggested that PthA4 has a higher affinity to the *PthA4‐EBE* (*EBE15*; score 4.95) when compared to the *PthA3213‐EBE* (*EBE3*; score: 10.20) (Figure 6). Therefore, it was counterintuitive that most of the PthA4‐induced executor transcripts are initiated from the *PthA3213‐EBE* rather than the *PthA4‐EBE* (Figure 5). The unexpected transcript ratios may be due to technical peculiarities of the RACE approach, where reverse transcription of mRNAs followed by PCR amplification favours the identification of shorter transcripts. For example, *PthA3213‐EBE‐* and *PthA4‐EBE*‐derived RACE products are 539 and 945 nucleotides long, respectively, potentially favouring amplification of short *PthA3213‐EBE*‐derived transcripts versus the longer *PthA4‐EBE*‐derived transcripts.

Previously, similar RACE studies have been conducted for a derivative of the rice executor gene *Xa27*, where the promoter was equipped with six *EBEs* matching to *Xoo* and *Xoc* TALE proteins (Hummel *et al*., 2012). These RACE studies suggested that six different TALEs, for which designated *pEBEs* had been integrated into the *Xa27* promoter, induced transcripts with mostly identical TSS rather than executor transcripts initiated by each TALE at its designated high‐affinity *pEBE* (Hummel *et al*., 2012). Similarly, RACE studies for a set of six distinct TALEs, designed to activate the rice *OsSULTR3; 6* gene identified only for two of six TALEs transcripts that were initiated ~50 nucleotides downstream of corresponding *EBEs* (Wang *et al*., 2017).

The discrepancies between our results and those of previously published studies may be explained, at least in part, by the different promoter contexts in which the *EBEs* were embedded. Alternatively, the studied rice genes might have TALE‐dependent next to TALE‐independent (leaky) transcription, which could possibly explain why some of the observed transcripts are not initiated from TALE‐predicted *EBEs*.

In summary, our RACE‐based studies of our *Xcc‐TALE‐trap* suggest that TALEs target preferentially code‐predicted *EBEs* to initiate executor transcripts. However, it also seems that RACE‐based analysis of TALE‐induced executor transcripts with distinct 5′ UTRs may not provide a perfect reflection, but rather an approximation of the *EBE* preferences of a given TALE.

### The *Xcc‐TALE‐trap* provides broad spectrum and potentially durable resistance to CBC

When we conceptually designed the *Xcc‐TALE‐trap*, the aim was to create an *R* gene that would mediate both broad spectrum and durable resistance to citrus canker. Indeed, our studies suggest that the *Xcc‐TALE‐trap* mediates broad‐spectrum resistance since inoculation of a collection of *Xcc* strains originating from four different continents showed that all strains trigger HR in the transgenic line containing the *Xcc‐TALE‐trap*, but not in the progenitor cultivar Duncan grapefruit (Figure 5). We also have reason to believe that the *Xcc‐TALE‐trap* will be durable based on the observation that most *Xcc* strains have multiple *TALE* genes each independently triggering HR (Figures 1 and 3). Therefore, a given *Xcc* strain must mutate several *TALE* genes simultaneously to escape detection by the *Xcc‐TALE‐trap*. This is in contrast to previously generated *EBE*‐depleted pathogen‐resistant plants, where transcriptional activation of a particular *S* gene via one newly evolved TALE that binds an alternative upstream *EBE* is likely to be sufficient to regain host compatibility (Nowack *et al*., 2022). We therefore assume that promoter traps mediating recognition of multiple different TALEs are more likely to confer durable resistance than host *S* gene derivatives in which the *EBEs* for single TALEs have been mutated.

Given that the TALE DNA‐binding domain is known to evolve rapidly (Nowack *et al*., 2022), we wondered to what extent newly evolved eTALEs from *Xcc* would be sensed by our *Xcc‐TALE‐trap*. Four recently identified eTALEs that evolved *in planta* from the *CsLOB1*‐activating progenitor PthA4 enabled us to address this question (Teper and Wang, 2021). The DNA binding domains of these eTALEs and their precursor PthA4 are similar but distinct (Figure S8). In this context, it is noteworthy that the *Xcc‐TALE‐trap* contains not only a high‐affinity *pEBE* for the PthA4 protein, but also seven additional *pEBEs* designed to recognize seven native *Xcc* TALEs (PthB, PthC, PthAw, PthA*, PthA, Apl2 and PthA 3213; Figure 1b) that are predicted to bind the same target region in the *CsLOB1* promoter as PthA4 by similar but distinct TALE repeat arrays. We therefore hypothesized that the functional collective of eight sequence‐related *EBEs* in the *Xcc‐TALE‐trap* would have a high probability to sense PthA4‐derived eTALEs. Indeed, our RACE studies somewhat support this hypothesis since in three out of four cases the eTALE‐induced executor transcripts are the consequence of interaction with either the *PthA4 pEBE* (*EBE15*) or the sequence‐related *PthA 3213 pEBE* (*EBE3*; Figure 7; Figures S7 and S8). In summary, our data suggest that TALE traps containing multiple sequence‐related *EBEs* have the potential to sense *in planta* evolving eTALEs with novel DNA binding domains, thereby conferring more durable resistance to *Xcc*. We anticipate that future studies will improve our understanding of the evolution of TALE DNA‐binding domains and may inspire improved trap designs that better anticipate changes in the TALE DNA‐binding domain. In summary, our results suggest that the availability of the TALE code, combined with the mechanistic understanding of the evolution of the TALE DNA‐binding domain, makes it possible to construct TALE‐specific *R* genes that confer effective protection not only against currently existing *Xcc* TALEs, but presumably also against newly evolving TALE proteins.

## Experimental procedures

### Construction of the binary vector for citrus transformation

Binary vector pTLAB21 containing the *Xcc‐TALE‐trap* was modified as follows: *avrGf2* gene (Gochez *et al*., 2015) with *Sac*I site was amplified and ligated into vector pK7Bs3_14EBE_:*avrGf1‐35S*‐terminator replacing *avrGf1*. The *Bs3* promoter carrying 14 EBEs was published previously (Shantharaj *et al*., 2017). The Bs3_14EBE_: avrGf1‐35S‐terminator was amplified with primers ATCCGGAATTCATATGACATGTTC‐TAATAAACGCTCTTTTCT containing *EcoR*I, *Nde*I, and reverse primer having *EcoR*I, ATCCGGAATTCCCAT‐GGCATGCTGGCTCCTTCAACGTTGCGG. The amplicon was ligated into pCR™/GW/TOPO (Life technologies, Carlsbad, California) with *EcoR*I site. The *35S* terminator was amplified with primers GGAATTCCATATGAGTCCGCAAAAATCACCA and GGAATTCCATATGTCACTGGATTTTGGTTT with *Nde*I site and ligated in front of *Bs3*_*14EBE*_. Cloned amplicons were amplified using Advantage HD Polymerase (Clontech, Palo Alto, California). Orientation and sequence was verified by Sanger sequencing. The *35S terminator‐ProBs3*_*14EBE*_ *:avrGf2‐35S* terminator sequence from pCR™/GW/TOPO was moved into pTLAB21 with *EcoR*I site. For plant transformation, Agrobacterium strain EHA101 (C58, rif; pTiBo542DT‐DNA, kan) was transformed with plasmid pTLAB21 by electroporation. EHA101 transconjugants were selected on streptomycin 50 mg/mL, kanamycin 50 mg/mL, and rifamycin 25 mg/mL.

### Transient *avrGf2* expression

The engineered binary construct with *Xcc‐TALE‐trap* was assayed for HR induction transiently by Agrobacterium on intact grapefruit leaves. A suspension of the *A. tumefaciens* EHA101 harbouring the binary construct was adjusted to OD_600_ = 0.3 and infiltrated into citrus leaves. Five hours later the infiltrated areas were infiltrated with *Xcc306* suspensions adjusted to 5 × 10^8^ cfu/mL. Plants were kept in the growth room at 28 °C, 12 h day/12 h night photoperiod and RH of 60% and inspected for HR symptoms.

### Production of transgenic citrus plants

Agrobacterium‐mediated transformation of Duncan grapefruit was carried out as previously described (Orbović and Grosser, 2007). Transgenic shoots that sprouted from explants co‐incubated with Agrobacterium were selected based on the GFP fluorescence. They were micrografted *in vitro* onto ‘Carrizo’ citrange [*Citrus sinensis* (L.) Osb. x *Poncirus trifoliata* (L.) Raf.] rootstock and later acclimatized by transferring to sterile soil. Once they reached the height of 25–30 cm, they were moved to the greenhouse and transferred to 15 cm diameter pots to obtain larger shoots. Genomic DNA was isolated from leaves and tested for *ProBs3*_*14EBE*_ *:avrGf2* by PCR. Transgenic plant material that tested positive was multiplied by grafting. Grafted plants were acclimatized in the greenhouse and tested for canker resistance.

### Plant pathogenicity assays

The experiments were conducted in the greenhouse environmental conditions at ambient air temperature at 25 °C day/21 °C night with humidity 60% day/night. Pathogenicity towards *Xcc306* was determined by inoculating leaves of transgenic and wild‐type Duncan grapefruit by bacterial suspension using four methods: (i) Infiltration assay in which leaves were infiltrated with bacterial suspensions adjusted to 10^8^ cfu/mL and the leaves were observed for HR daily for 4 days. (ii) Population growth assay where bacterial suspension was adjusted to 10^5^ cfu/mL and infiltrated using a hypodermic needle and syringe. Leaf discs of 1 cm^2^ were harvested at 0, 2, 4, 6, and 8 days after infiltration with a cork borer and then ground in 1 mL sterile tap water. The homogenate was serially diluted and plated on NA plates. Colonies were counted 48 h after plating. (iii) A leaf pin prick assay was done by placing a drop of bacterial suspension (10^8^ cfu/mL) on the adaxial leaf surface and then the leaf was pinpricked through the drop with a syringe and hypodermic needle. Leaves were inspected for canker lesions at a regular time interval. (iv) A spray inoculation test in which the plants were misted with a bacterial suspension adjusted to 10^8^ cfu/mL and bagged for 48 h. Plants were monitored for a regular period for canker lesion appearance.

### Quantitative real‐time PCR

Leaves of transgenic grapefruit carrying the *Xcc‐TALE‐trap* were infiltrated with either sterile tap water (mock), or bacterial suspensions (*Xcc306* or *Xcc306*Δ4) prepared in sterile tap water. Suspensions were adjusted to an OD_600_ = 0.3 (~5 × 10^8^ cfu/mL). The experiment consisted of two replicates consisting of two leaf discs (1 cm^2^ leaf tissue), which were collected and flash‐frozen in liquid nitrogen at 0, 24, and 48 h post‐inoculation (h p.i.). Samples were powdered using a 1600 MiniG™ SPEX sample prep machine and RNA was extracted using TRI Reagent (Sigma Chemical Company, St. Louis, Missouri) according to the manufacturer's protocol. Following extraction, RNA samples were treated with DNase (TURBO DNA‐free™ kit; Invitrogen by Thermo Fisher Scientific, Waltham, Massachusetts). A total quantity of 600 ng of RNA was used per sample to synthesize cDNA by reverse transcription using the ProtoScript II First Strand cDNA Synthesis Kit (New England Biolabs, Ipswich, Massachusetts) with the anchored oligo‐d(T) primer [d(T)23VN]. Real‐time PCR was carried out on a CFX96™ Real‐Time System (Bio‐Rad Laboratories, Berkeley, California) using SsoFast™ EvaGreen Supermix and 2 μL of 1:10 diluted cDNA template. Each experimental sample was replicated three times. Amplicons were subjected to melting curve analysis (65–95 °C in 0.5 °C increments). PCR for *avrGf2* was accomplished using the primer set qavrGf2F (Table S4). PCR of citrus *Ef1a* was used as constitutive standard (Table S4). The expression data were analysed by relative quantification normalized to a reference gene using 2^−DDCT^.

### Rapid amplification of cDNA ends

5′ RACE was carried out on transgenic grapefruit inoculated with transconjugants containing individual TALEs or eTALEs. Suspensions were adjusted to an OD_600_ of 0.3 (5 × 10^8^ cfu/mL). Plants were kept in the greenhouse. For each sample, 2 cm^2^ leaf discs were collected and flash‐frozen in liquid nitrogen 48 h after infiltration. Samples were powdered using a 1600 MiniG™ SPEX sample prep machine and total RNA was extracted using TRI Reagent (Sigma Chemical Company) according to manufacturer's protocol. Following extraction, RNA samples were treated with DNase (TURBO DNA‐free™ kit, Invitrogen by Thermo Fisher Scientific). A total quantity of 1000 ng of RNA was used per sample to synthesis 5′‐RACE‐ready cDNA using the SMARTer RACE 5′/3′ kit (Takara Bio USA, Inc., Mountain View, CA) following the kit protocol. Primary 5′‐RACE PCR reactions and nested PCR were accomplished following kit guidelines using *avrGf2* gene‐specific primers (GSPs) GSP‐150, GSP249, and GSP589 (Table S4). RACE products were cloned into the pRACE vector and sequenced using the M13F universal primer.

### Comparing *Xcc‐TALE‐trap* transgenic and non‐transgenic Duncan grapefruit under field conditions

Nine *Xcc*‐*TALE‐trap* transgenic citrus trees and seven wild‐type Duncan grapefruit trees were planted at a USDA facility in Fort Pierce, Florida on 28 March 2019 in a randomized design. Plants were spaced 76 cm apart. Following transplanting of these trees into the field, trees were typically sprayed every 2 weeks for insect control only. Trees were exposed to natural inoculum from citrus canker‐infected trees. Trees were rated for disease severity on 5 June, 27 September, and 3 December 2019. The disease severity ratings were based on a scale from 1 to 4 (1: no visible canker symptoms, 2: a few lesions, 3: prevalent lesions on multiple leaves and 4: many lesions on individual leaves and widely distributed on the tree). Statistical analysis of disease ratings at each time point was conducted using the nonparametric Wilcoxon rank‐sum test in PROC NPAR1WAY of SAS (SAS Institute, Cary, NC).

### Accession numbers

The nucleotide sequence of pTLab21, a T‐DNA plasmid containing the *Xcc‐TALE‐trap* has been deposited at GenBank under the accession number: OQ601558.

## Conflicts of interest

The authors declare no conflict of interest.

## Author contributions

D.S. carried out all experimental work, unless otherwise stated, and prepared the first draft of the article. G.V.M. carried out RACE studies. V.O. carried out the transformation of grapefruit plants. D.H. and P.R. designed the *EBE* assembly of the *Xcc‐TALE‐trap*. D.R.H carried out data and statistical analysis related to Figures 3 and 4; Figure S3 and edited the article. T.L. was involved in the conceptual design of the *Xcc‐TALE‐trap* and prepared a revised version of the article figures and text. J.J. conceived the idea, supervised the experimental studies, coordinated the project, and assisted in drafting and finalizing the manuscript. All authors read the article and approved the final version.

## Supporting information


**Figure S1** An *avrGf2*‐based promoter trap triggers cell death in grapefruit only when being inoculated together with *Xcc306*, a *Xanthomonas* strain containing TALEs matching to the promoter trap.
**Figure S2** A diagnostic PCR shows an *avrGf2*‐specific amplification product on template DNA from one plant of several putative transgenic grapefruit plants.
**Figure S3** In transgenic grapefruit lines containing the *Xcc‐TALE‐trap*, *Xcc306* TALE proteins transcriptionally activate both the *avrGf2* executor transgene as well as the *CsLOB1* endogene.
**Figure S4** A promoter trap with tandem‐arranged *EBEs* mediates recognition of four distinct TALE proteins from *Xcc306*.
**Figure S5** A transgenic grapefruit line, with a promoter trap, designed to recognize *Xcc* TAL effectors, does not show HR upon delivery of TAL effectors from *X. oryzae* pv. *oryzae*.
**Figure S6** *Xcc* strains from different continents all have *TALE* genes.
**Figure S7** Location of transcriptional start sites (TSS) of *avrGf2* executor transcripts within the *Xcc‐TALE‐trap*.
**Figure S8** Evolved PthA4‐derived TALEs transcriptionally activating the CsLOB1 promoter and the *Xcc‐TALE‐trap*.

**Table S1** Collection of *Xcc* strains of distinct geographical origin that were inoculated into leaves of Duncan grapefruit and a derived transgenic line containing the *Xcc‐TALE‐trap*.
**Table S2** The *Xcc‐TALE‐trap* confers resistance to citrus canker in a field study.
**Table S3** Features of engineered executor *R* genes.
**Table S4** List of primers used in this study.

## References

[pbi14109-bib-0001] Antony, G. , Zhou, J. , Huang, S. , Li, T. , Liu, B. , White, F. and Yang, B. (2010) Rice *xa13* recessive resistance to bacterial blight is defeated by induction of the disease susceptibility gene *Os‐11N3* . Plant Cell, 22, 3864–3876. 21098734 10.1105/tpc.110.078964 PMC3015117

[pbi14109-bib-0002] Bonas, U. , Stall, R.E. and Staskawicz, B. (1989) Genetic and structural characterization of the avirulence gene *avrBs3* from *Xanthomonas campestris* pv. *vesicatoria* . Mol. Gen. Genet. 218, 127–136. 2550761 10.1007/BF00330575

[pbi14109-bib-0003] Conti, G. , Xoconostle‐Cazares, B. , Marcelino‐Perez, G. , Hopp, H.E. and Reyes, C.A. (2021) Citrus genetic transformation: an overview of the current strategies and insights on the new emerging technologies. Front. Plant Sci. 12, 768197. 34917104 10.3389/fpls.2021.768197 PMC8670418

[pbi14109-bib-0004] Doyle, E.L. , Booher, N.J. , Standage, D.S. , Voytas, D.F. , Brendel, V.P. , Vandyk, J.K. and Bogdanove, A.J. (2012) TAL effector‐nucleotide targeter (TALE‐NT) 2.0: tools for TAL effector design and target prediction. Nucleic Acids Res. 40, W117–W122. 22693217 10.1093/nar/gks608 PMC3394250

[pbi14109-bib-0005] Girbig, M. , Misiaszek, A.D. and Müller, C.W. (2022) Structural insights into nuclear transcription by eukaryotic DNA‐dependent RNA polymerases. Nat. Rev. Mol. Cell Biol. 23, 603–622. 35505252 10.1038/s41580-022-00476-9

[pbi14109-bib-0006] Gochez, A.M. , Minsavage, G.V. , Potnis, N. , Canteros, B.I. , Stall, R.E. and Jones, J.B. (2015) A functional XopAG homologue in *Xanthomonas fuscans* pv. *aurantifolii* strain C limits host range. Plant Pathol. 64, 1207–1214.

[pbi14109-bib-0007] Gochez, A.M. , Shantharaj, D. , Potnis, N. , Zhou, X. , Minsavage, G.V. , White, F.F. , Wang, N. *et al*. (2017) Molecular characterization of XopAG effector AvrGf2 from *Xanthomonas fuscans* ssp. *aurantifolii* in grapefruit. Mol. Plant Pathol. 18, 405–419. 27030294 10.1111/mpp.12408 PMC6638233

[pbi14109-bib-0008] Hu, Y. , Zhang, J. , Jia, H. , Sosso, D. , Li, T. , Frommer, W.B. , Yang, B. *et al*. (2014) *Lateral organ boundaries 1* is a disease susceptibility gene for citrus bacterial canker disease. Proc. Natl Acad. Sci. USA, 111, E521–E529. 24474801 10.1073/pnas.1313271111 PMC3910620

[pbi14109-bib-0009] Huang, X. , Wang, Y. and Wang, N. (2021) Highly efficient generation of canker‐resistant sweet orange enabled by an improved CRISPR/Cas9 system. Front. Plant Sci. 12, 769907. 35087548 10.3389/fpls.2021.769907 PMC8787272

[pbi14109-bib-0010] Huang, X. , Wang, Y. and Wang, N. (2022) Base editors for citrus gene editing. Front. Genome Ed. 4, 852867. 35296063 10.3389/fgeed.2022.852867 PMC8919994

[pbi14109-bib-0011] Hummel, A.W. , Doyle, E.L. and Bogdanove, A.J. (2012) Addition of transcription activator‐like effector binding sites to a pathogen strain‐specific rice bacterial blight resistance gene makes it effective against additional strains and against bacterial leaf streak. New Phytol. 195, 883–893. 22747776 10.1111/j.1469-8137.2012.04216.x

[pbi14109-bib-0012] Ji, C. , Ji, Z. , Liu, B. , Cheng, H. , Liu, H. , Liu, S. , Yang, B. *et al*. (2020) *Xa1* allelic *R* genes activate rice blight resistance suppressed by interfering TAL effectors. Plant Commun. 1, 100087. 33367250 10.1016/j.xplc.2020.100087 PMC7748017

[pbi14109-bib-0013] Ji, Z. , Sun, H. , Wei, Y. , Li, M. , Wang, H. , Xu, J. , Lei, C. *et al*. (2022) Ectopic expression of executor gene *Xa23* enhances resistance to both bacterial and fungal diseases in rice. Int. J. Mol. Sci. 23, 6545. 35742990 10.3390/ijms23126545 PMC9224217

[pbi14109-bib-0014] Jia, H. , Orbovic, V. , Jones, J.B. and Wang, N. (2016) Modification of the PthA4 effector binding elements in Type I *CsLOB1* promoter using Cas9/sgRNA to produce transgenic Duncan grapefruit alleviating *Xcc*ΔpthA4:dCsLOB1.3 infection. Plant Biotechnol. J. 14, 1291–1301. 27071672 10.1111/pbi.12495 PMC11389130

[pbi14109-bib-0015] Jia, H.G. , Zhang, Y.Z. , Orbovic, V. , Xu, J. , White, F.F. , Jones, J.B. and Wang, N. (2017) Genome editing of the disease susceptibility gene *CsLOB1* in citrus confers resistance to citrus canker. Plant Biotechnol. J. 15, 817–823. 27936512 10.1111/pbi.12677 PMC5466436

[pbi14109-bib-0016] Jia, H. , Omar, A.A. , Orbović, V. and Wang, N. (2022a) Biallelic editing of the *LOB1* Promoter via CRISPR/Cas9 creates canker‐resistant ‘Duncan’ grapefruit. Phytopathology, 112, 308–314. 34213958 10.1094/PHYTO-04-21-0144-R

[pbi14109-bib-0017] Jia, H. , Wang, Y. , Su, H. , Huang, X. and Wang, N. (2022b) LbCas12a‐D156R efficiently edits *LOB1* effector binding elements to generate canker‐resistant citrus plants. Cell, 11, 315. 10.3390/cells11030315 PMC8834406 35159125

[pbi14109-bib-0018] Kay, S. , Hahn, S. , Marois, E. , Hause, G. and Bonas, U. (2007) A bacterial effector acts as a plant transcription factor and induces a cell size regulator. Science, 318, 648–651. 17962565 10.1126/science.1144956

[pbi14109-bib-0019] Li, Z. , Lifang, Z. , Gang, Y. , Li, X. , Zhiyuan, J. , Muhammad, Z. , Ni, H. *et al*. (2014) A potential disease susceptible gene *CsLOB* of citrus is targeted by a major virulence effector PthA of *Xanthomonas citri* subsp. *citri* . Mol. Plant, 7, 912–915. 24398629 10.1093/mp/sst176

[pbi14109-bib-0020] Maruta, N. , Burdett, H. , Lim, B.Y.J. , Hu, X. , Desa, S. , Manik, M.K. and Kobe, B. (2022) Structural basis of NLR activation and innate immune signalling in plants. Immunogenetics, 74, 5–26. 34981187 10.1007/s00251-021-01242-5 PMC8813719

[pbi14109-bib-0021] Naqvi, S.A.H. , Wang, J. , Malik, M.T. , Umar, U.‐U.‐D. , Ateeq Ur, R. , Hasnain, A. , Sohail, M.A. *et al*. (2022) Citrus canker ‐ distribution, taxonomy, epidemiology, disease cycle, pathogen biology, detection, and management: a critical review and future research agenda. Agronomy, 12, 1075.

[pbi14109-bib-0022] Nowack, M.K. , Holmes, D.R. and Lahaye, T. (2022) TALE‐induced cell death executors: an origin outside immunity? Trends Plant Sci. 27, 536–548. 34924289 10.1016/j.tplants.2021.11.003 PMC7612725

[pbi14109-bib-0023] Orbović, V. and Grosser, J.W. (2007) Citrus. In Agrobacterium Protocols, Vol. 2 ( Wang, K. , ed), pp. 177–189. Totowa, NJ: Humana Press.

[pbi14109-bib-0024] Peng, A.H. , Chen, S.C. , Lei, T.G. , Xu, L.Z. , He, Y.R. , Wu, L. , Yao, L.X. *et al*. (2017) Engineering canker‐resistant plants through CRISPR/Cas9‐targeted editing of the susceptibility gene CsLOB1 promoter in citrus. Plant Biotechnol. J. 15, 1509–1519. 28371200 10.1111/pbi.12733 PMC5698050

[pbi14109-bib-0025] Plaschka, C. , Hantsche, M. , Dienemann, C. , Burzinski, C. , Plitzko, J. and Cramer, P. (2016) Transcription initiation complex structures elucidate DNA opening. Nature, 533, 353–358. 27193681 10.1038/nature17990

[pbi14109-bib-0026] Read, A.C. , Hutin, M. , Moscou, M.J. , Rinaldi, F.C. and Bogdanove, A.J. (2020a) Cloning of the rice *Xo1* resistance gene and interaction of the Xo1 protein with the defense‐suppressing *Xanthomonas* effector Tal2h. Mol. Plant Microbe Interact. 33, 1189–1195. 32748677 10.1094/MPMI-05-20-0131-SC

[pbi14109-bib-0027] Read, A.C. , Moscou, M.J. , Zimin, A.V. , Pertea, G. , Meyer, R.S. , Purugganan, M.D. , Leach, J.E. *et al*. (2020b) Genome assembly and characterization of a complex zfBED‐NLR gene‐containing disease resistance locus in carolina gold select rice with nanopore sequencing. PLoS Genet. 16, e1008571. 31986137 10.1371/journal.pgen.1008571 PMC7004385

[pbi14109-bib-0028] Roeschlin, R.A. , Uviedo, F. , Garcia, L. , Molina, M.C. , Favaro, M.A. , Chiesa, M.A. , Tasselli, S. *et al*. (2019) PthA4(AT), a 7.5‐repeats transcription activator‐like (TAL) effector from *Xanthomonas citri* ssp. *citri*, triggers citrus canker resistance. Mol. Plant Pathol. 20, 1394–1407. 31274237 10.1111/mpp.12844 PMC6792138

[pbi14109-bib-0029] Römer, P. , Hahn, S. , Jordan, T. , Strauß, T. , Bonas, U. and Lahaye, T. (2007) Plant‐pathogen recognition mediated by promoter activation of the pepper *Bs3* resistance gene. Science, 318, 645–648. 17962564 10.1126/science.1144958

[pbi14109-bib-0030] Römer, P. , Recht, S. and Lahaye, T. (2009a) A single plant resistance gene promoter engineered to recognize multiple TAL effectors from disparate pathogens. Proc. Natl Acad. Sci. USA, 106, 20526–20531. 19910532 10.1073/pnas.0908812106 PMC2776607

[pbi14109-bib-0031] Römer, P. , Strauss, T. , Hahn, S. , Scholze, H. , Morbitzer, R. , Grau, J. , Bonas, U. *et al*. (2009b) Recognition of AvrBs3‐like proteins is mediated by specific binding to promoters of matching pepper *Bs3* alleles. Plant Physiol. 150, 1697–1712. 19448036 10.1104/pp.109.139931 PMC2719119

[pbi14109-bib-0032] Rybak, M. , Minsavage, G.V. , Stall, R.E. and Jones, J.B. (2009) Identification of *Xanthomonas citri* ssp. *citri* host specificity genes in a heterologous expression host. Mol. Plant Pathol. 10, 249–262. 19236573 10.1111/j.1364-3703.2008.00528.x PMC6640320

[pbi14109-bib-0033] Schornack, S. , Ballvora, A. , Gürlebeck, D. , Peart, J. , Baulcombe, D. , Baker, B. , Ganal, M. *et al*. (2004) The tomato resistance protein Bs4 is a predicted non‐nuclear TIR‐NB‐LRR protein that mediates defense responses to severely truncated derivatives of AvrBs4 and overexpressed AvrBs3. Plant J. 37, 46–60. 14675431 10.1046/j.1365-313x.2003.01937.x

[pbi14109-bib-0034] Shantharaj, D. , Römer, P. , Figueiredo, J.F.L. , Minsavage, G.V. , Krönauer, C. , Stall, R.E. , Moore, G.A. *et al*. (2017) An engineered promoter driving expression of a microbial avirulence gene confers recognition of TAL effectors and reduces growth of diverse *Xanthomonas* strains in citrus. Mol. Plant Pathol. 18, 976–989. 27362693 10.1111/mpp.12454 PMC6638256

[pbi14109-bib-0035] Sharma, A. , Abrahamian, P. , Carvalho, R. , Choudhary, M. , Paret, M.L. , Vallad, G.E. and Jones, J.B. (2022) Future of bacterial disease management in crop production. Annu. Rev. Phytopathol. 60, 259–282. 35790244 10.1146/annurev-phyto-021621-121806

[pbi14109-bib-0036] Strauß, T. , Van Poecke, R. , Strauß, A. , Römer, P. , Minsavage, G.V. , Singh, S. , Wolf, C. *et al*. (2012) RNA‐seq pinpoints a *Xanthomonas* TAL‐effector activated resistance gene in a large crop genome. Proc. Natl Acad. Sci. USA, 109, 19480–19485. 23132937 10.1073/pnas.1212415109 PMC3511116

[pbi14109-bib-0037] Streubel, J. , Baum, H. , Grau, J. , Stuttmann, J. and Boch, J. (2017) Dissection of TALE‐dependent gene activation reveals that they induce transcription cooperatively and in both orientations. PLoS One, 12, e0173580. 28301511 10.1371/journal.pone.0173580 PMC5354296

[pbi14109-bib-0038] Teper, D. and Wang, N. (2021) Consequences of adaptation of TAL effectors on host susceptibility to *Xanthomonas* . PLoS Genet. 17, e1009310. 33465093 10.1371/journal.pgen.1009310 PMC7845958

[pbi14109-bib-0039] Teper, D. , White, F.F. and Wang, N. (2023) The dynamic transcription activator‐like effector family of *Xanthomonas* . Phytopathology, 113, 651–666. 36449529 10.1094/PHYTO-10-22-0365-KD

[pbi14109-bib-0040] Tian, D. , Wang, J. , Zeng, X. , Gu, K. , Qiu, C. , Yang, X. , Zhou, Z. *et al*. (2014) The rice TAL effector‐dependent resistance protein XA10 triggers cell death and calcium depletion in the endoplasmic reticulum. Plant Cell, 26, 497–515. 24488961 10.1105/tpc.113.119255 PMC3963592

[pbi14109-bib-0041] Tran, T.T. , Perez‐Quintero, A.L. , Wonni, I. , Carpenter, S.C.D. , Yu, Y. , Wang, L. , Leach, J.E. *et al*. (2018) Functional analysis of African *Xanthomonas oryzae* pv. *oryzae* TALomes reveals a new susceptibility gene in bacterial leaf blight of rice. PLoS Pathog. 14, e1007092. 29864161 10.1371/journal.ppat.1007092 PMC6037387

[pbi14109-bib-0042] Triplett, L.R. , Cohen, S.P. , Heffelfinger, C. , Schmidt, C.L. , Huerta, A. , Tekete, C. , Verdier, V. *et al*. (2016) A resistance locus in the American heirloom rice variety carolina gold select is triggered by TAL effectors with diverse predicted targets and is effective against African strains of *Xanthomonas oryzae* pv. *oryzicola* . Plant J. 87, 472–483. 27197779 10.1111/tpj.13212 PMC5030141

[pbi14109-bib-0043] Wang, C. , Zhang, X. , Fan, Y. , Gao, Y. , Zhu, Q. , Zheng, C. , Qin, T. *et al*. (2015) XA23 is an executor R protein and confers broad‐spectrum disease resistance in rice. Mol. Plant, 8, 290–302. 25616388 10.1016/j.molp.2014.10.010

[pbi14109-bib-0044] Wang, L. , Rinaldi, F.C. , Singh, P. , Doyle, E.L. , Dubrow, Z.E. , Tran, T.T. , Perez‐Quintero, A.L. *et al*. (2017) TAL effectors drive transcription bidirectionally in plants. Mol. Plant, 10, 285–296. 27965000 10.1016/j.molp.2016.12.002

[pbi14109-bib-0045] Wu, B. , Yu, Q. , Deng, Z. , Duan, Y. , Luo, F. and Gmitter, F., Jr. (2022) A chromosome‐level phased genome enabling allele‐level studies in sweet orange: a case study on citrus Huanglongbing tolerance. Hortic. Res. 10, uhac247. 36643761 10.1093/hr/uhac247 PMC9832951

[pbi14109-bib-0046] Yuan, M. , Ke, Y. , Huang, R. , Ma, L. , Yang, Z. , Chu, Z. , Xiao, J. *et al*. (2016) A host basal transcription factor is a key component for infection of rice by TALE‐carrying bacteria. Elife, 5, e19605. 27472897 10.7554/eLife.19605 PMC4993585

[pbi14109-bib-0047] Zeng, X. , Tian, D. , Gu, K. , Zhou, Z. , Yang, X. , Luo, Y. , White, F.F. *et al*. (2015) Genetic engineering of the *Xa10* promoter for broad‐spectrum and durable resistance to *Xanthomonas oryzae* pv. *oryzae* . Plant Biotechnol. J. 13, 993–1001. 25644581 10.1111/pbi.12342

