# Supplementary material for: A promoter trap in transgenic citrus mediates recognition of a broad spectrum of Xanthomonas citri pv. citri TALEs, including in planta‐evolved derivatives
Source: Plant Biotechnol J. 2023 Jul 8;21(10):2019–32. doi: 10.1111/pbi.14109 (PMC10502743; doi:10.1111/pbi.14109)
Supplement: Supplementary file 1 — Figure S1 An avrGf2‐based promoter trap triggers cell death in grapefruit only when being inoculated together with Xcc306, a Xanthomonas strain containing TALEs matching to the promoter trap. Figure S2 A diagnostic PCR shows an avrGf2‐specific amplification product on template DNA from one plant of several putative transgenic grapefruit plants. Figure S3 In transgenic grapefruit lines containing the Xcc‐TALE‐trap, Xcc306 TALE proteins transcriptionally activate both the avrGf2 executor transgene as well as the CsLOB1 endogene. Figure S4 A promoter trap with tandem‐arranged EBEs mediates recognition of four distinct TALE proteins from Xcc306. Figure S5 A transgenic grapefruit line, with a promoter trap, designed to recognize Xcc TAL effectors, does not show HR upon delivery of TAL effectors from X. oryzae pv. oryzae. Figure S6 Xcc strains from different continents all have TALE genes. Figure S7 Location of transcriptional start sites (TSS) of avrGf2 executor transcripts within the Xcc‐TALE‐trap. Figure S8 Evolved PthA4‐derived TALEs transcriptionally activating the CsLOB1 promoter and the Xcc‐TALE‐trap. [file PBI-21-2019-s001.docx]

# Supplementary Tables

| Table S1: Collection of *Xcc* strains of distinct geographical origin that were inoculated into leaves of Duncan grapefruit and a derived transgenic line containing the *Xcc*-TALE-trap. | | |
| --- | --- | --- |
|  | ***Xcc* strains** | |
| **No.** | **designation** | **origin (continent)** |
| 1 | *Xcc* 306 ΔpthA1-4 (*Xcc*Δ1-4) | Brazil (South America) |
| 2 | *Xcc* 306 | Brazil (South America) |
| 3 | *Xcc* A44 | Argentina (South America) |
| 4 | *Xcc* 2004-00059 | Florida (North America) |
| 5 | *Xcc* 46 | India (Asia) |
| 6 | *Xcc* 82 | Japan (Asia) |
| 7 | *Xcc* 101 | Guam (Oceania) |
| 8 | *Xcc* 106 | Australia (Oceania) |
| 9 | *Xcc* 111 | China (Asia) |
| 10 | *Xcc* 131 | Maldives Island (Asia) |
| 11 | *Xcc* 126 | Korea (Asia) |
| 12 | *Xcc* 275-2 | Thailand (Asia) |
| *Xanthomonas citri* ssp. *citri* A group strains were inoculated 10^8^ cfu/mL. Leaves were analyzed for elicitation of HR or canker pathogenicity symptoms. | | |

| Table S2. The *Xcc*-TALE-trap confers resistance to citrus canker in a field study. | | | |
| --- | --- | --- | --- |
|  | Disease ratings on: | | |
| Citrus genotype | June 5^th^ | September 27^th^ | December 2^nd^ |
| Duncan grapefruit | 1.1^a^ | 1.7*^b^ | 2.6** |
| *Xcc*-TALE-trap | 1.0 | 1.0 | 1.0 |
| Comparison of *Xcc*-TALE-trap transgenic Duncan citrus and corresponding wild-type Duncan grapefruit plants for citrus canker susceptibility under field conditions studied in 2019 in Fort Pierce, Florida, USA.  ^a^Value represents disease ratings: 1 = no visible symptoms; 2 = a few lesions; 3 = prevalent lesions on multiple leaves; and 4 = many lesions on individual leaves and widely distributed.  ^b^Asterisks indicate significant differences between Duncan grapefruit and the transgenic line containing the *Xcc*-TALE-trap as analyzed by Wilcoxon rank sum test where * and ** represent significantly different values at p < 0.05 and p < 0.01, respectively. | | | |

| Table S3: Features of engineered executor *R* genes | | | | | | |
| --- | --- | --- | --- | --- | --- | --- |
| Publication (designation of the promoter trap) | Promoter scaffold (origin);  & transgenic plant | Executor gene (origin) | *EBEs* added | *EBE*-spacing | Intra-*EBE* homology | RACE data |
| Zeng [2015] Plant Biotechnol J 13 (Xa10^E5^) | *Xa10* (rice); rice | *Xa10* (rice) | 5 | 3-9 | No | No |
| Hummel [2012] New Phytol 195, 883 (UXO) | *Xa27* (rice); rice | *Xa27* (rice) | 6 | 5-11 | No | Yes |
| Hummel [2012] New Phytol 195, 883 (XOC) | *Xa27* (rice); rice | *Xa27* (rice) | 3 | 35-40 | No | No |
| Hummel [2012] New Phytol 195, 883 (XOO) | *Xa27* (rice); rice | *Xa27* (rice) | 3 | 34-36 | No | No |
| Wang [2018] Mol Plant Pathol 19, 2025 (-) | *Xa10* (rice); rice | *Bs4C* (pepper) | - | - | - | No |
| Gui [2022] Transgenic Res 31, 119 (-) | *Xa10* (rice); rice | Bax (mouse) | - | - | - | No |
| Hutin [2016] Plant J 88, 43 (-) | *pUAS_gal4_* (DEX-inducible); rice | AVR1-CO39 (*M. oryzae* effector) + Pi-CO39 (rice NLR) |  |  |  | No |
| Ji [2022] Int J Mol Sci 23, 6545 (Xa23p1.0) | *Xa23* (rice); rice | *Xa23* (rice) | 1 | - | - |  |
| Römer [2009] Proc Natl Acad Sci USA 106, 20526 (-) | *Bs3* (pepper);  *N. benthamiana** | *Bs3* (pepper) | 3 | 39-131 | No | Yes |
| Shantharaj [2023]  (*Xcc*-TALE-trap) | *Bs3* (pepper);  grapefruit | *avrGf2* (*Xanthomonas* effector) | 14 | 15 | Yes | Yes |
| * This executor trap was tested only in the context of transient assays and not in stable transgenic lines. | | | | | | |

| Table S4. List of primers used in this study | | |
| --- | --- | --- |
| 1 | designation | Nucleotide sequence |
| 2 | qavrGf2F | TACAACGTGGCAGCGTATATC |
| 3 | qavrGf2R | CGTTGTCGTAAACAGCGTTAAG |
| 4 | qef1aF | GGCTGATTGTGCTGTCCTTATC |
| 5 | qef1aR | CCAAGGGTGAAAGCAAGTAGAG |
| 6 | GSP150 | GATTACGCCAAGCTTGAACAAGAGGAGAAAAGAGAAAGGGGATG |
| 7 | GSP249 | GATTACGCCAAGCTTGACATAAGCAGCACCGGCAGTTCCCGCAACGT |
| 8 | GSP589 | GATTACGCCAAGCTTCAGTGAGTAGGGCGGGCTTTATACGCTCGCCA |
