# Supplementary material for: A promoter trap in transgenic citrus mediates recognition of a broad spectrum of Xanthomonas citri pv. citri TALEs, including in planta‐evolved derivatives
Source: Plant Biotechnol J. 2023 Jul 8;21(10):2019–32. doi: 10.1111/pbi.14109 (PMC10502743; doi:10.1111/pbi.14109)
Supplement: Supplementary file 2 — Table S1 Collection of Xcc strains of distinct geographical origin that were inoculated into leaves of Duncan grapefruit and a derived transgenic line containing the Xcc‐TALE‐trap. Table S2 The Xcc‐TALE‐trap confers resistance to citrus canker in a field study. Table S3 Features of engineered executor R genes. Table S4 List of primers used in this study. [file PBI-21-2019-s002.pdf]

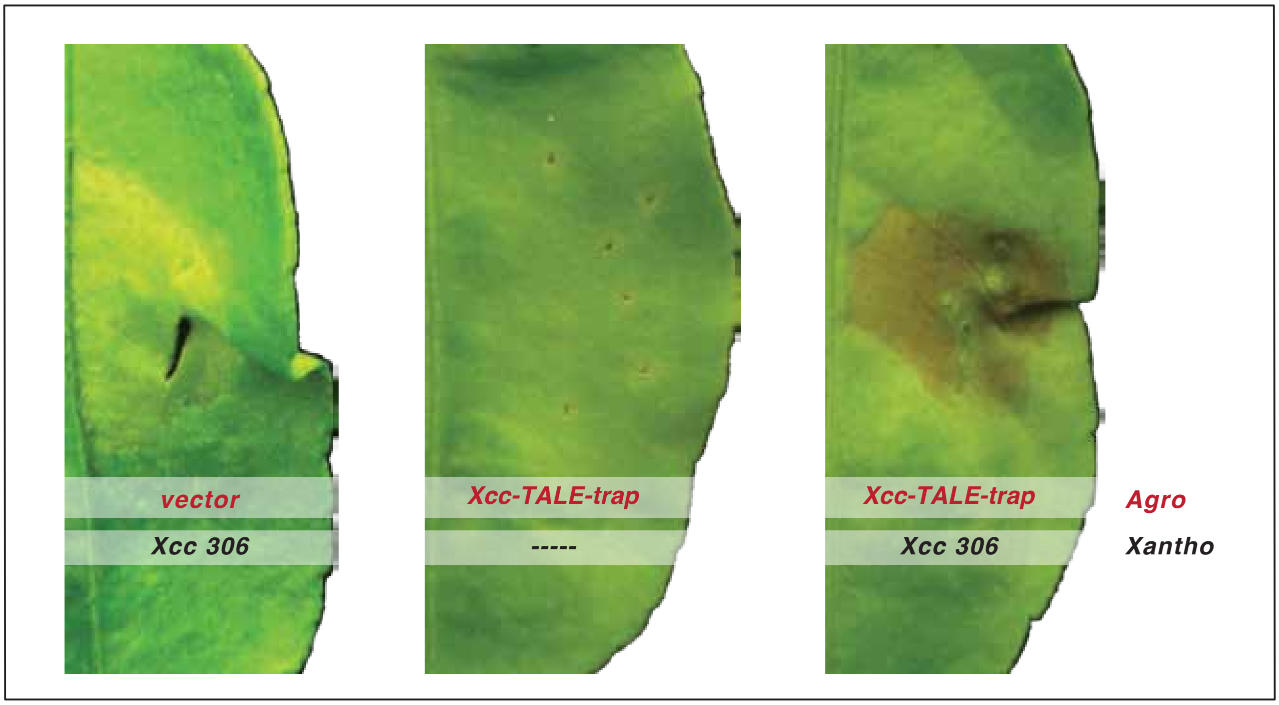

Figure S1 The *Xcc*-TALE-trap triggers cell death only when being inoculated together with *Xcc306*, a *Xanthomonas* strain containing TALEs compatible to *EBEs* present in the *Xcc-TALE-trap*. Agrobacteria (OD_{600} = 0.3) delivering either the *Xcc-TALE-trap* T-DNA or a vector control were inoculated into Duncan grapefruit leaves with or without *Xcc306* ($5x10^8$ cfu/ml), an *Xcc* strain containing TALEs compatible with *EBEs* in the promoter of the *Xcc-TALE-trap*. Notably, the *Xcc-TALE-trap* induces cell death only when co-inoculated with *Xcc306*. This suggests that cell death is TALE dependent. Transient inoculation assays were analyzed 4 days post inoculation.

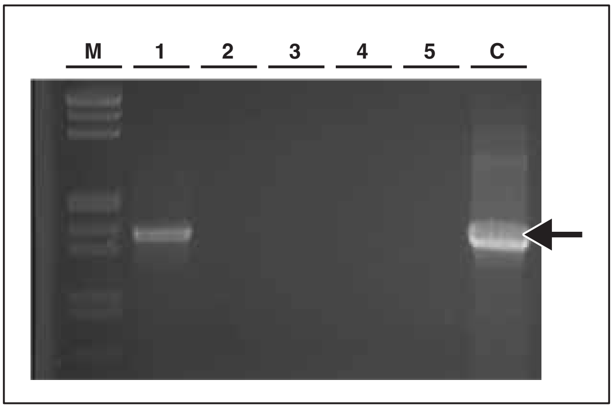

Figure S2 A diagnostic PCR shows an *avrGf2*-specific amplification product on template DNA from one plant of several putative transgenic grapefruit plants. GFP-expressing plant lines (lanes 1-5) were tested by PCR for presence of the *avrGf2* transgene. The arrow indicates a PCR fragment that was amplified from a plasmid template (control; C) containing the *AvrGf2* transgene. M, size marker.

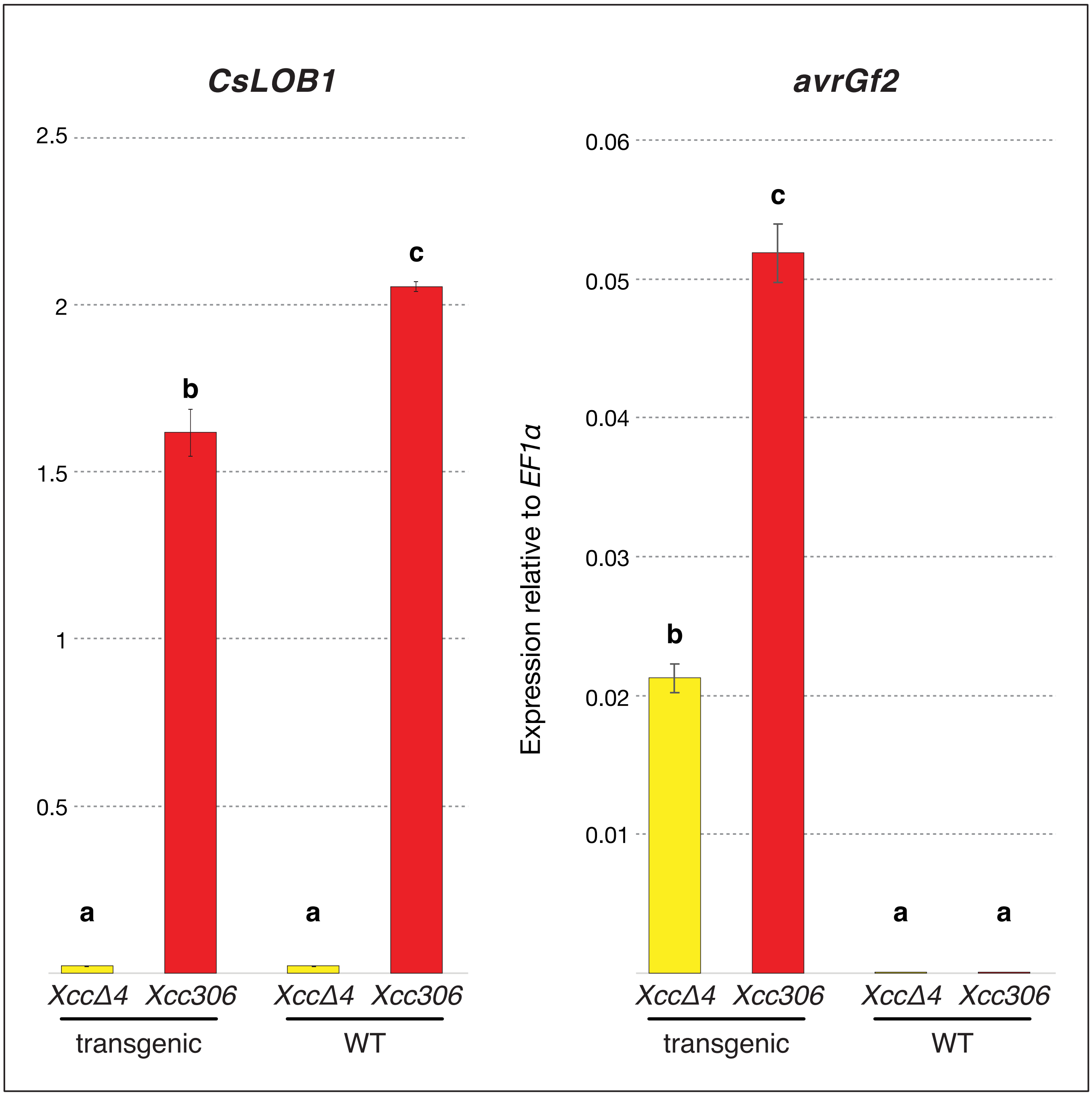

Figure S3 In transgenic grapefruit lines containing the *Xcc-TALE-trap*, *Xcc306* TALE proteins transcriptionally activate both the *avrGf2* executor transgene as well as the *CsLOB1* endogene. Gene expression was quantified at 48 hours post inoculation with either *Xcc306* (5x10^8 cfu/ml) or its mutant derivative *XccΔ4* in either the transgenic line or wildtype (WT) Duncan grapefruit. Indicated transcript levels were normalized to *EF1α*. Data are presented as bar graphs with the mean value of 4 technical replicates from one set of cDNA and error bars displaying the calculated standard deviation. Statistically significant groups ($p<0.01$) are indicated with lower-case letters calculated according to one-way ANOVA followed by a Tukey HSD post hoc test.

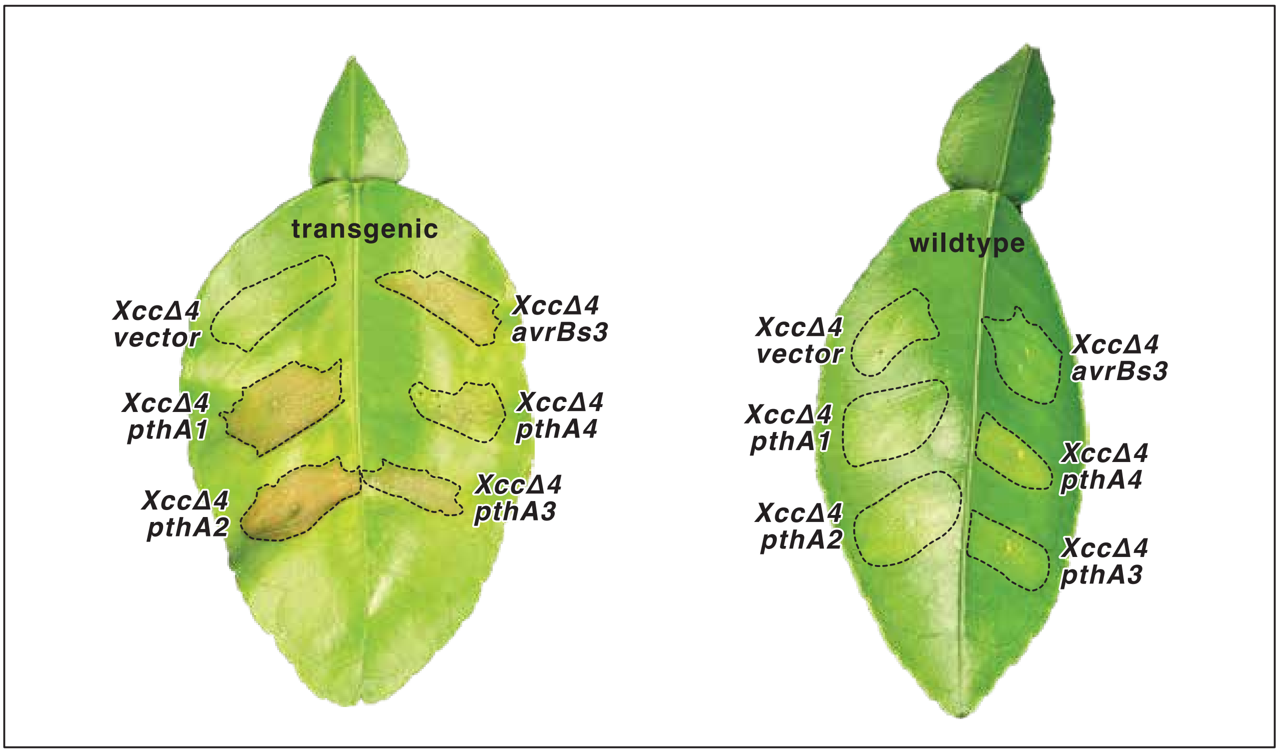

Figure S4 A promoter trap with tandem arranged *EBEs* mediates recognition of four distinct TALE proteins from *Xcc 306*. Leaves of Duncan grapefruit (citrus wildtype) and a derived transgenic line containing a promoter trap (transgenic), were inoculated with *XccΔ4* (*Xcc 306* mutant lacking *pthA1*, *pthA2*, *pthA3* and *pthA4*) or derived transconjugants containing PthA1 (*Xcc pthA1*), PthA2 (*Xcc pthA2*), PthA3 (*Xcc pthA1*), PthA4 (*Xcc pthA4*) or AvrBs3 (*Xcc avrBs3*). Inoculated leaf sections are indicated by dashed lines. Picture was taken at 4 days post inoculation. Bacteria were adusted to 5x10^8 cfu/ml before inoculation.

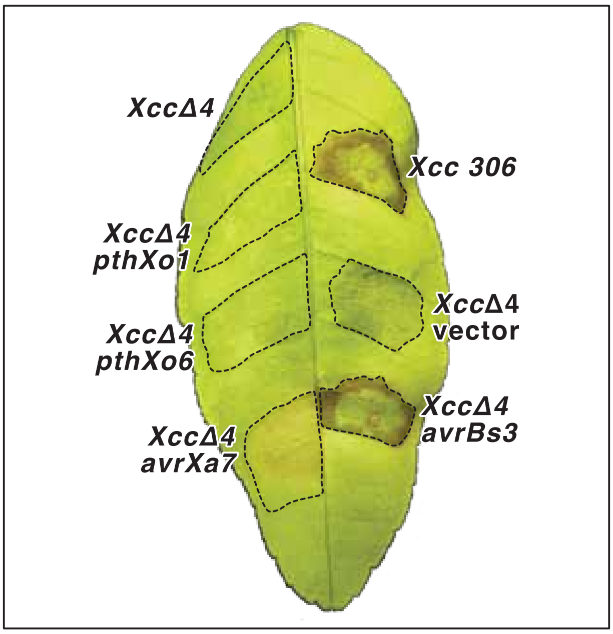

Figure S5 A transgenic grapefruit line, with a promoter trap, designed to recognize *Xcc* TAL effectors, does not show HR upon delivery of TAL effectors from *X. oryzae* pv. *oryzae*.The transgenic line was incoulated with the depicted *X. citri* strains (10^8 cfu/ml). Inoculated leaf sections are indicated by dashed lines. The picture was taken at 4 days post inoculation.

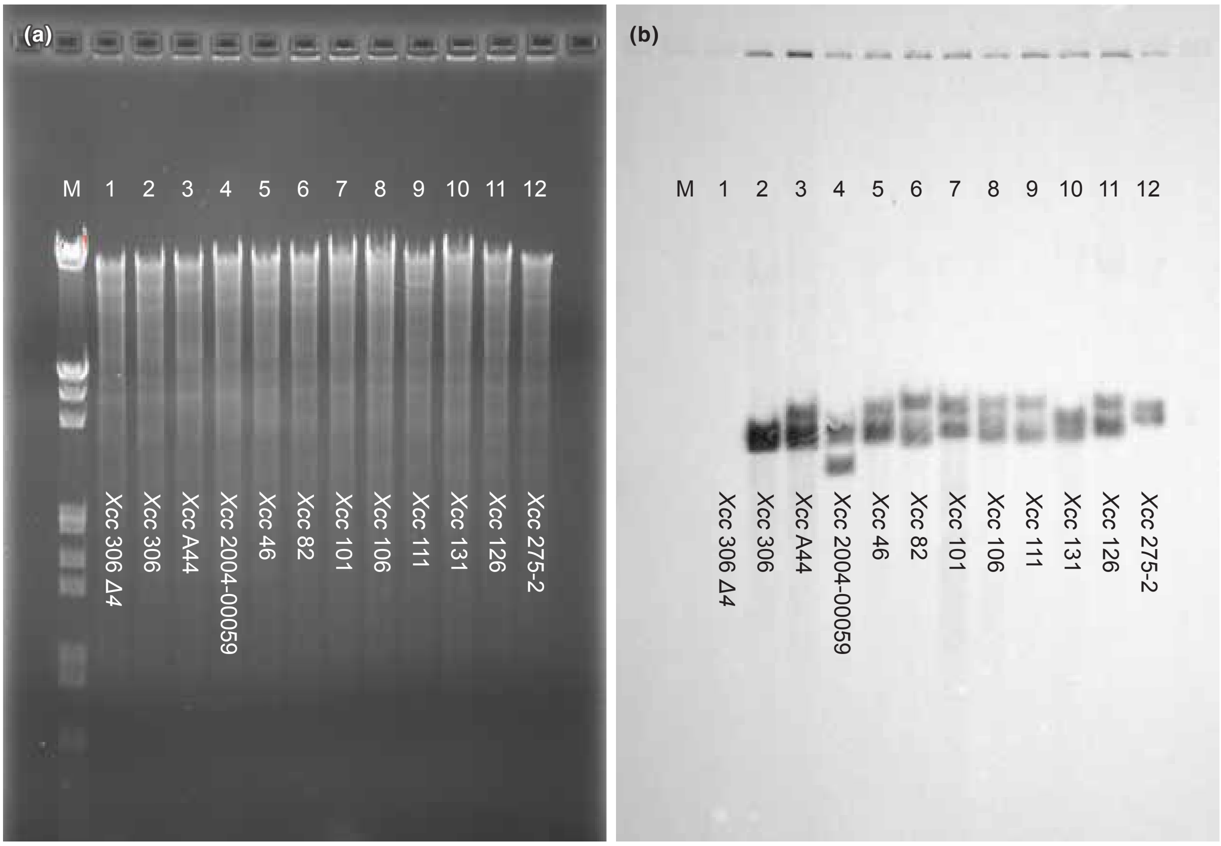

Figure S6 *Xcc* strains from different continents all have *TALE* genes. (a) Size-fractionated DNA from *Xcc* strains from different continents. Genomic DNA from the *Xcc* isolates shown was digested with BamHI and size-fractionated on a 1% agarose gel containing ethidium bromide. M indicates the DNA size standard (λ DNA/EcoRI + HindIII marker; Promega). (b) Southern analysis shows that *Xcc* strains from different continents all contain genes that hybridise to *pthA4 repeats*. The size-separated DNA shown in (a) was transferred to positively charged nylon membranes (Roche) for subsequent Southern analysis. The internal BamHI fragment of the *Xcc306 pthA4* gene was labelled for use as a probe using the DIG-High Prime DNA Labelling and Detection Starter Kit I (Roche). The hybridisation signal was visualised by colour detection using NBT/BCIP (Roche).

Figure S7 (a) 5' RACE data for PthA1

PthA1 - 5' RACE data for treated material / Race primer: gattacgccaagcttGACATAAGCAGCACCGGCAGTTCCCGCAACGTA

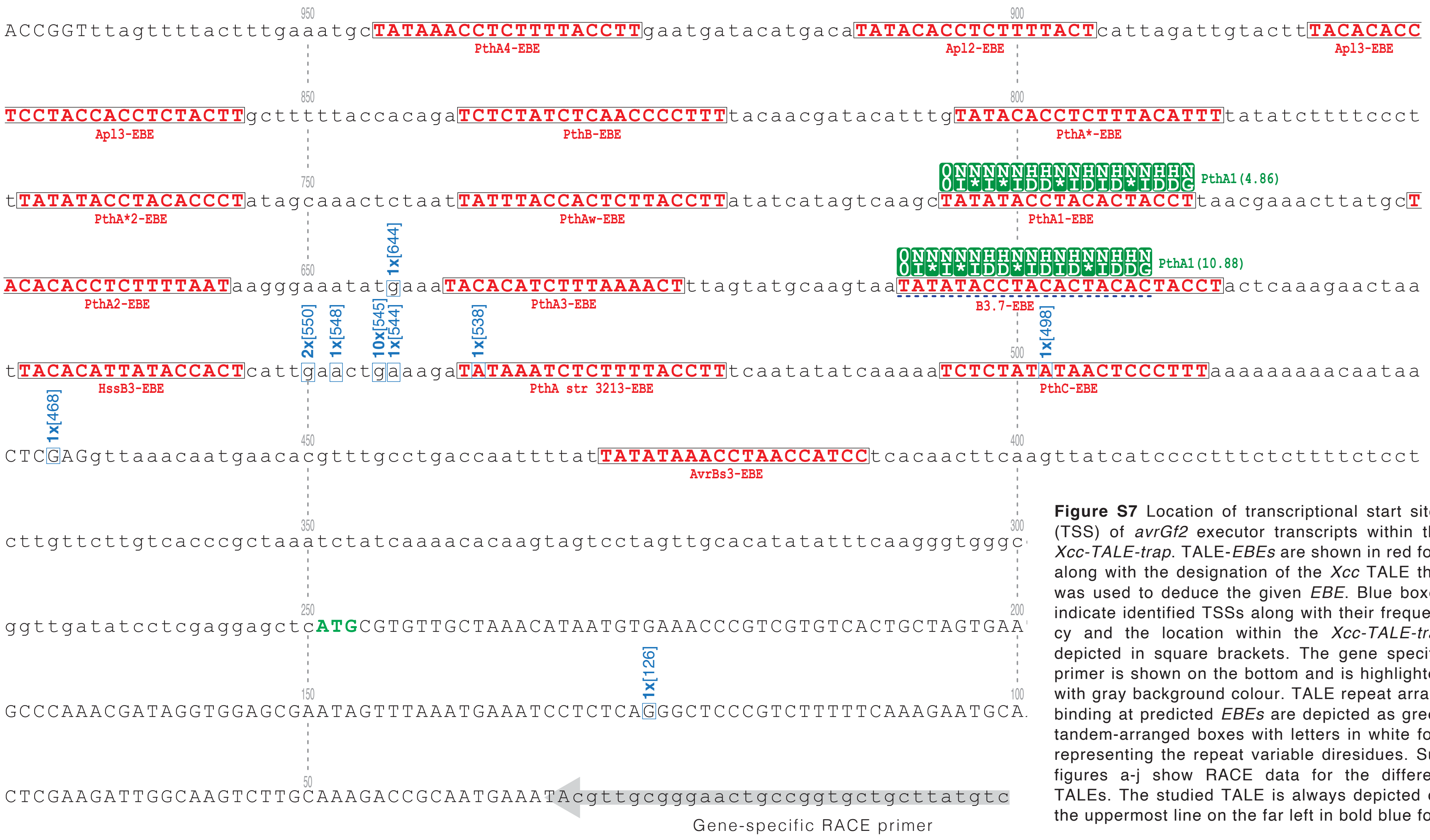

Figure S7 Location of transcriptional start sites (TSS) of *avrGf2* executor transcripts within the *Xcc-TALE-trap*. TALE-*EBEs* are shown in red font along with the designation of the *Xcc* TALE that was used to deduce the given *EBE*. Blue boxes indicate identified TSSs along with their frequency and the location within the *Xcc-TALE-trap* depicted in square brackets. The gene specific primer is shown on the bottom and is highlighted with gray background colour. TALE repeat arrays binding at predicted *EBEs* are depicted as green tandem-arranged boxes with letters in white font representing the repeat variable diresidues. Sub figures a-j show RACE data for the different TALEs. The studied TALE is always depicted on the uppermost line on the far left in bold blue font.

Figure S7 (b) 5’ RACE data for PthA2

PthA2 - 5’ RACE data for treated material / Race primer: gattacgccaagcttGACATAAGCAGCACCGGCAGTTCCCGCAACGTA

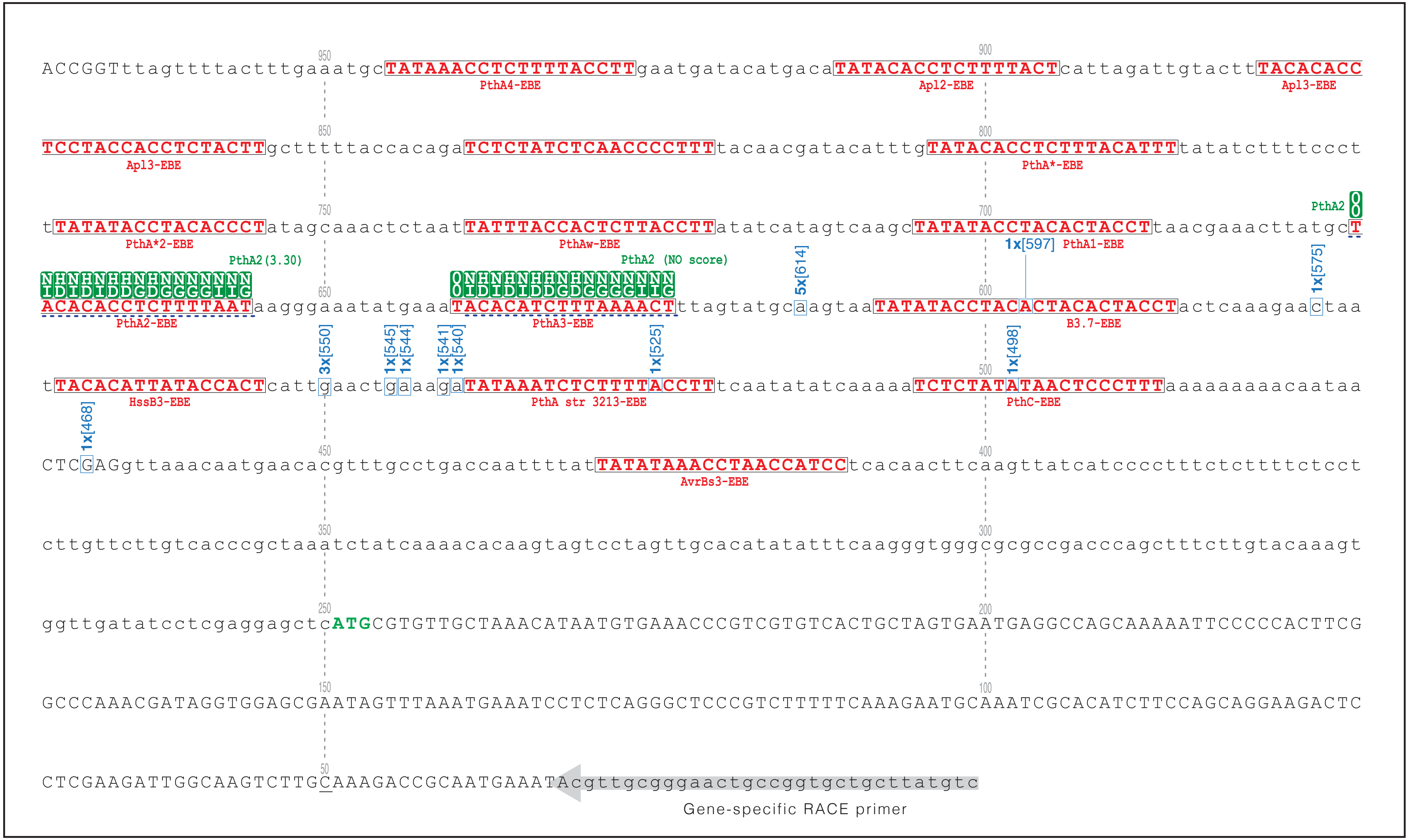

Figure S7 (c) 5’ RACE data for PthA3

PthA3 - 5’ RACE data for treated material / Race primer: gattacgccaagcttGACATAAGCAGCACCGGCAGTTCCCGCAACGTA

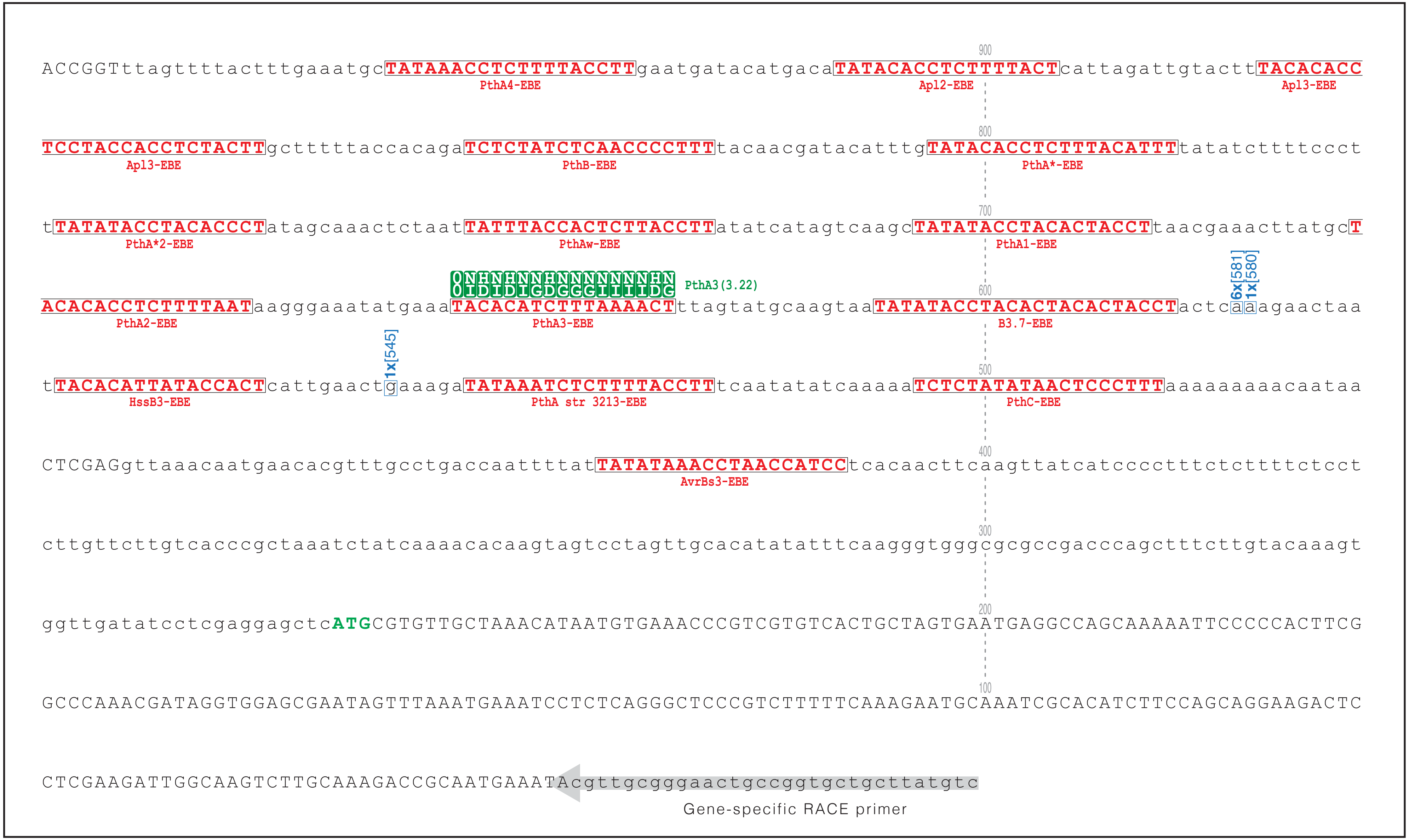

Figure S7 (d) 5’ RACE data for PthA4

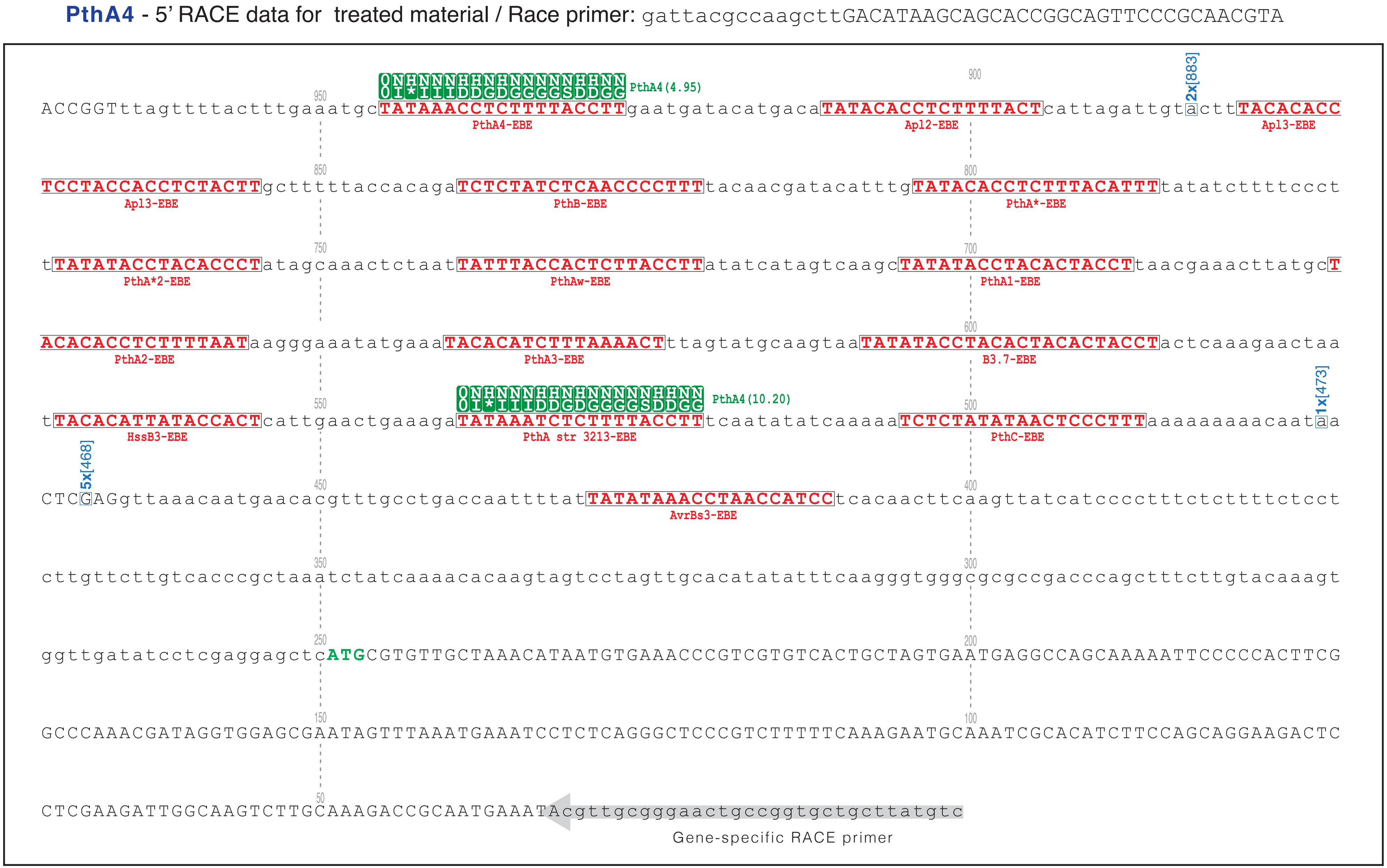

Figure S7 (e) 5’ RACE data for AvrBs3

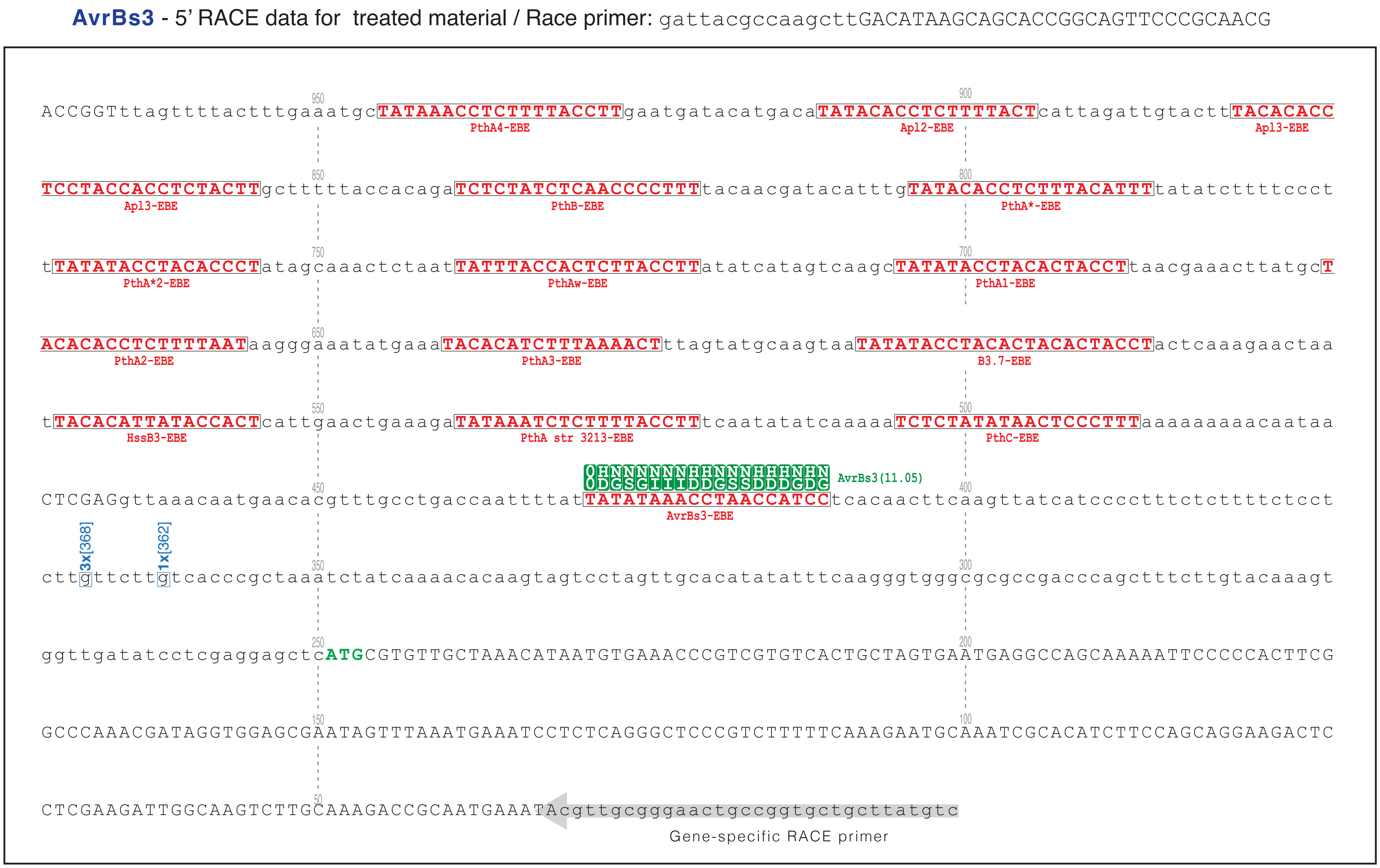

Figure S7 (f) 5' RACE data for dTALELB2A1

dTALELB2A1 - 5' RACE data for treated material / Race primer: GAACAAGAGGAGAAAAGAGAAAGGGGATG

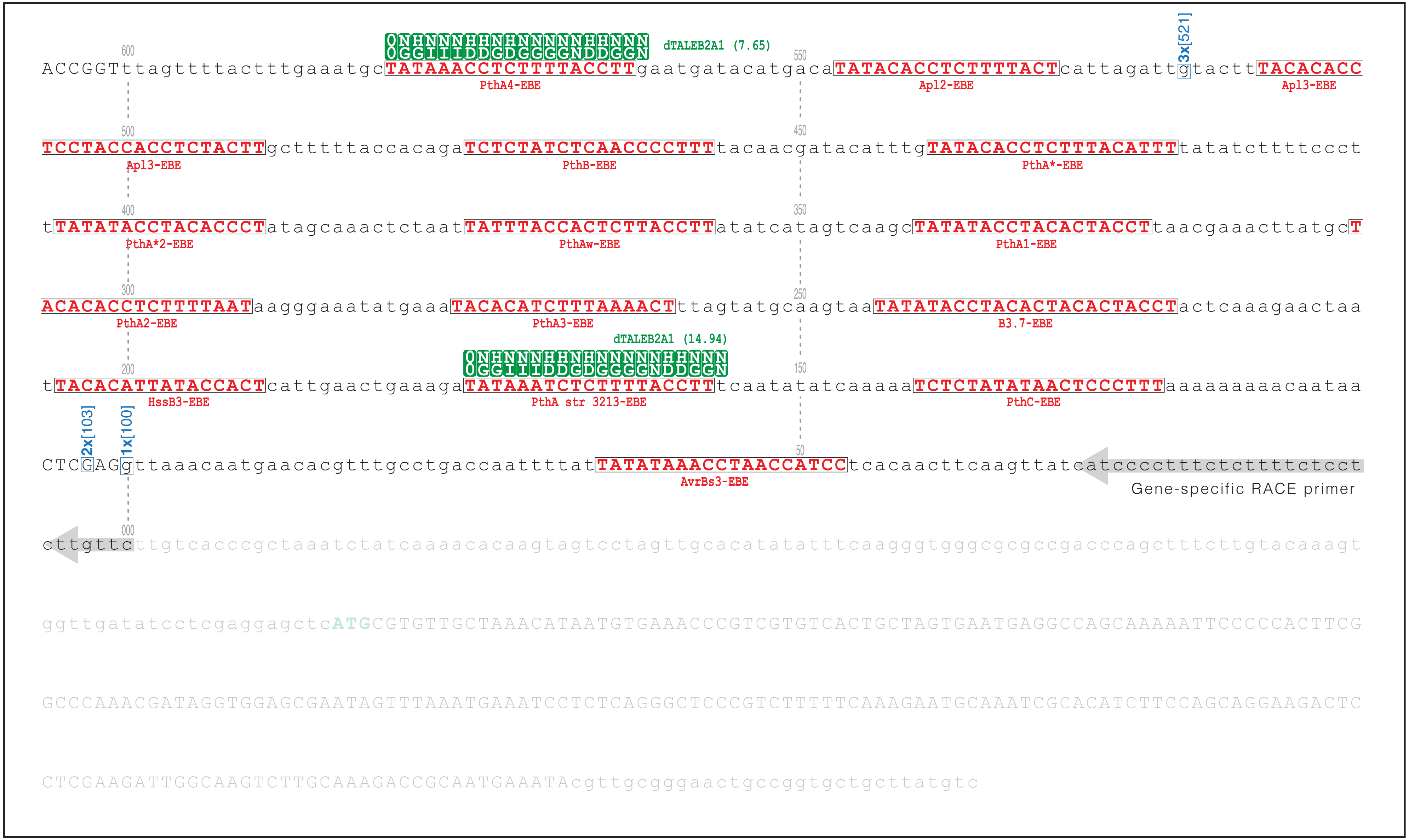

Figure S7 (g) 5’ RACE data for dTALELB2A2

dTALELB2A2 - 5’ RACE data for treated material / Race primer: GAACAAGAGGAGAAAAGAGAAAGGGGATG

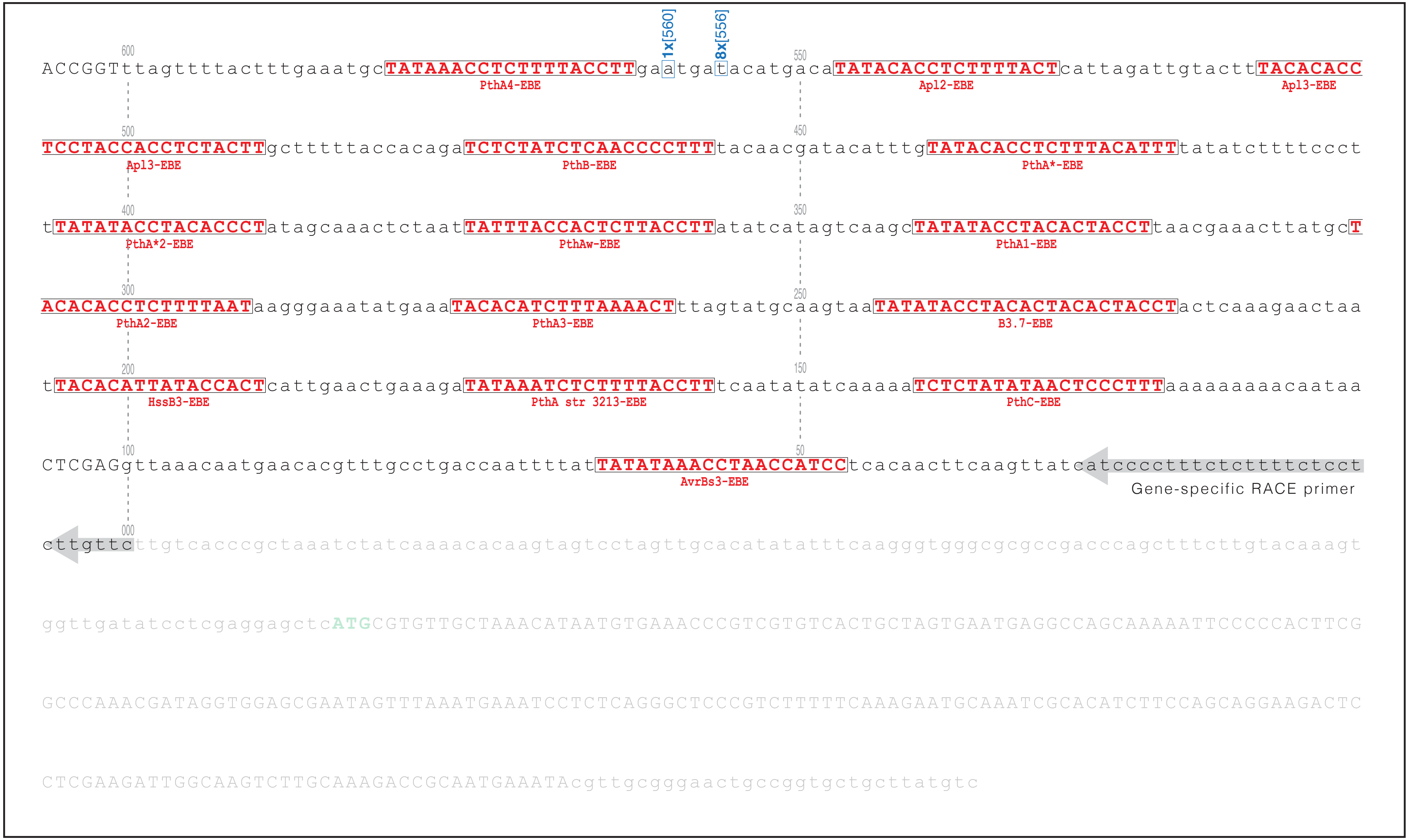

Figure S7 (h) 5’ RACE data for dTALELB3A

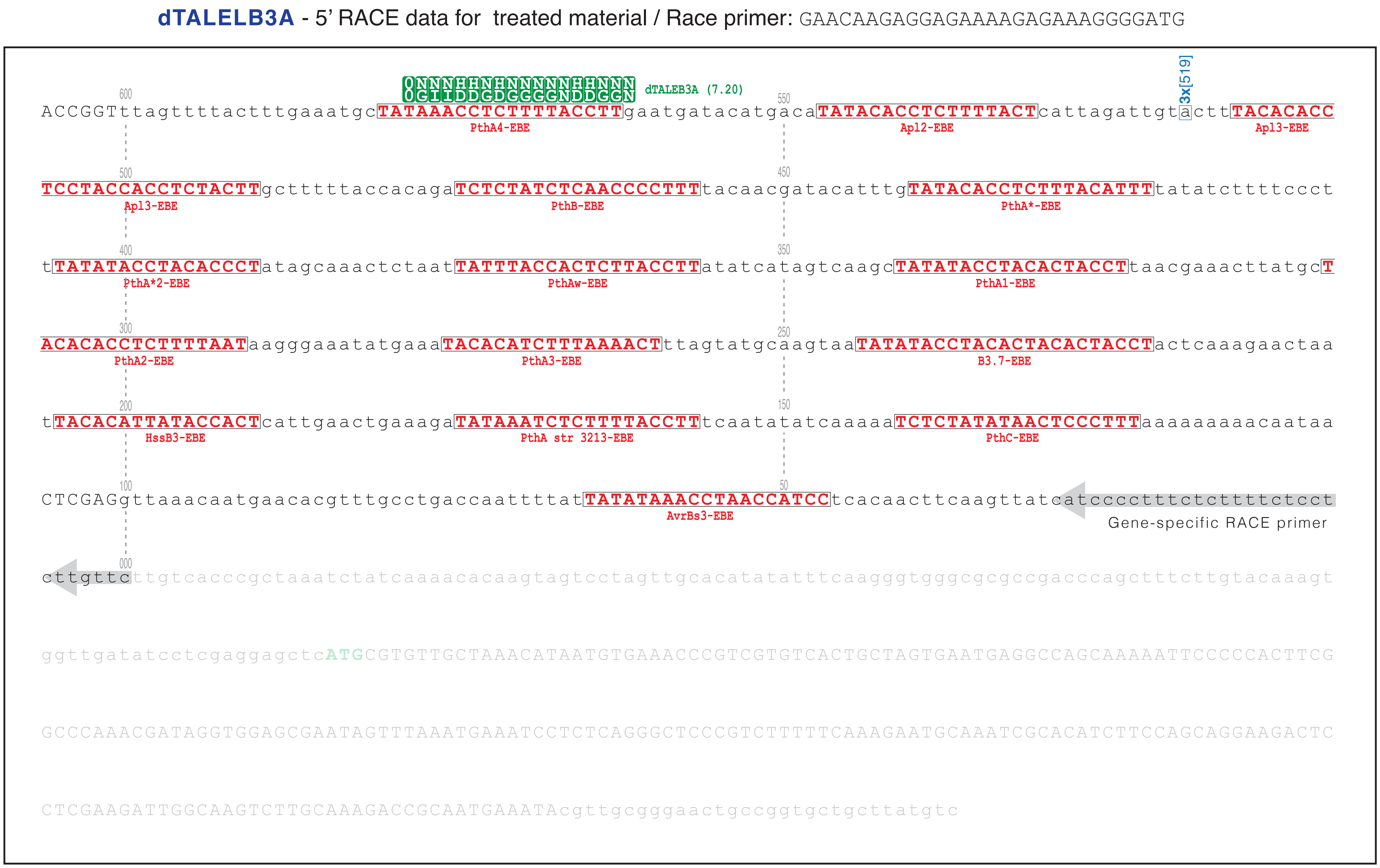

Figure S7 (i) 5' RACE data for dTALELB5A

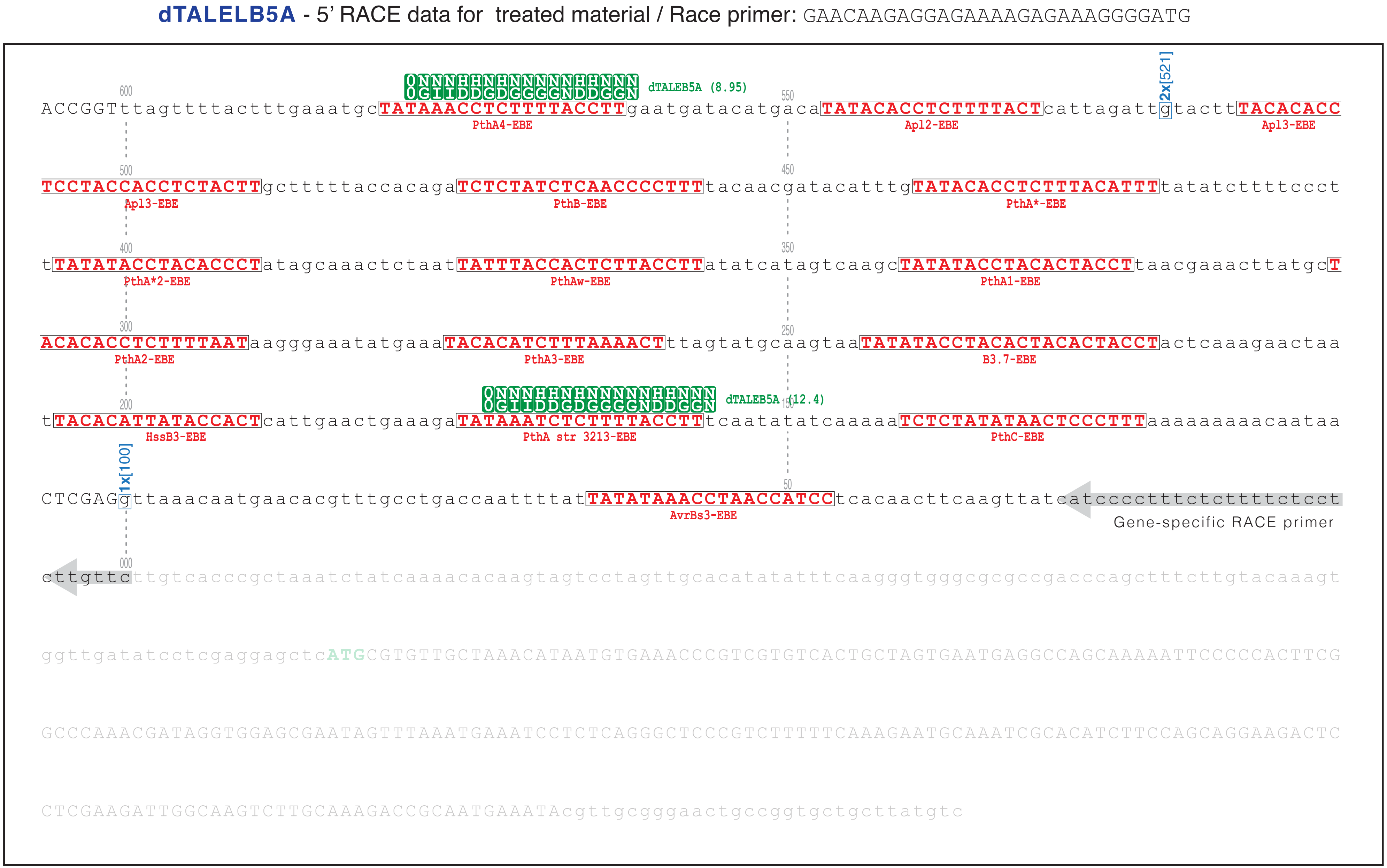

Figure S7 (j) 5' RACE data for dTALEWTLOB1

dTALEWTLOB1 - 5' RACE data for treated material / Race primer: GAACAAGAGGAGAAAAGAGAAAGGGGATG

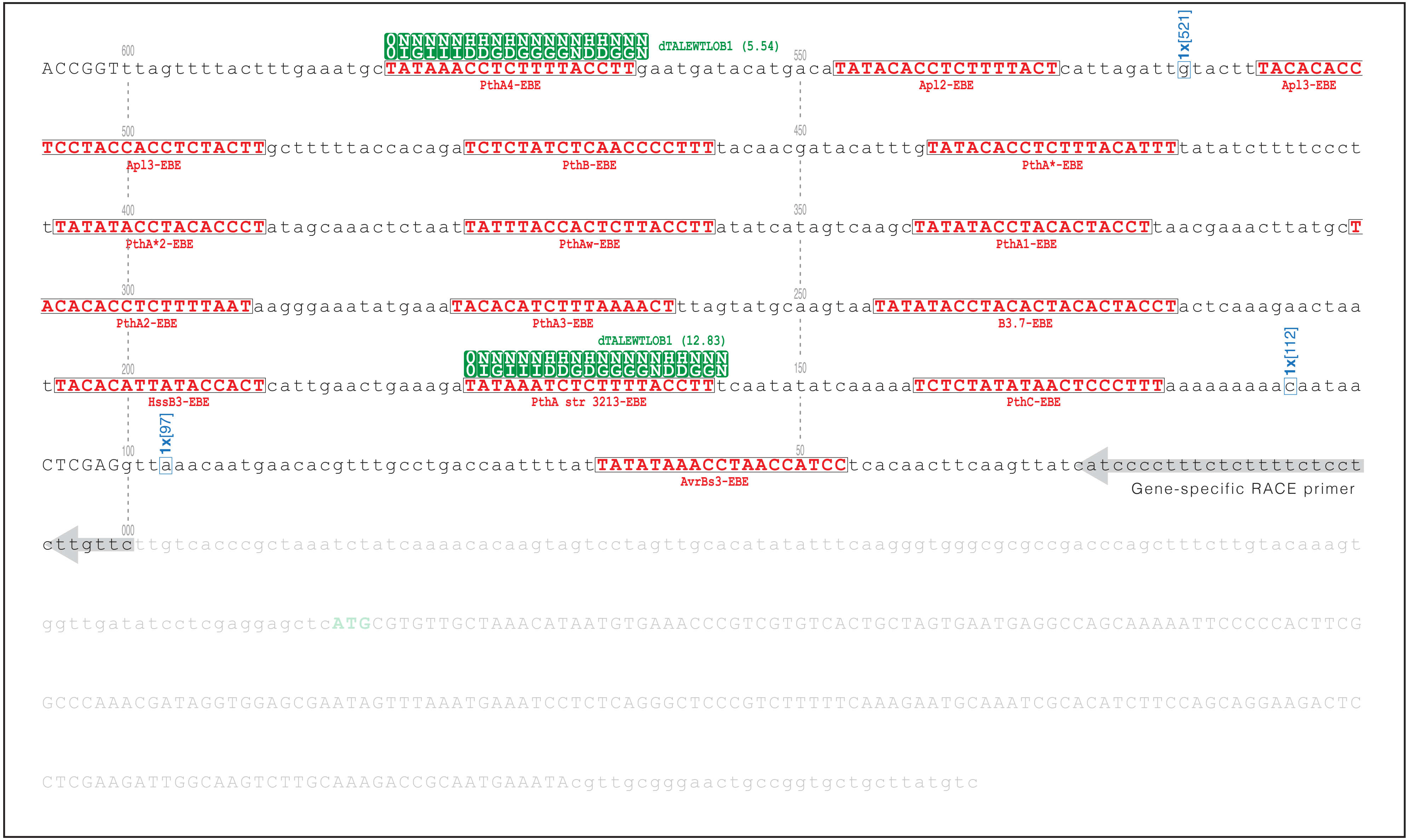

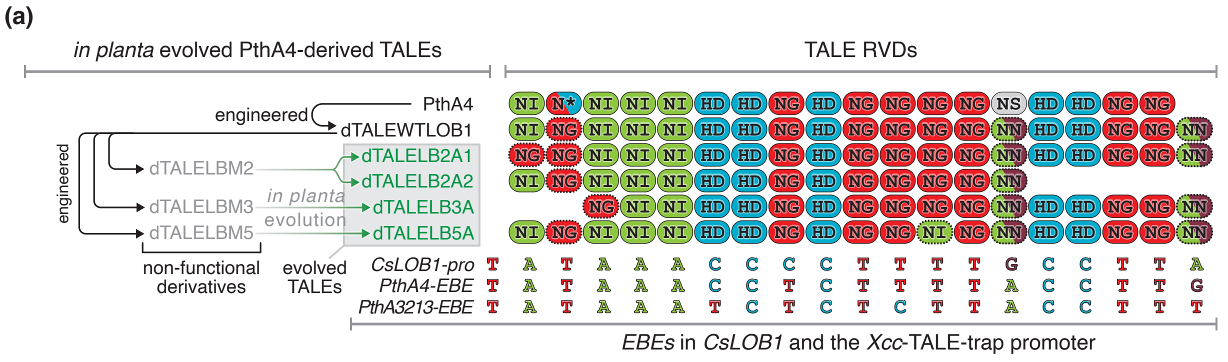

(b)

TALE	PthA4-*EBE**	PthA3213-*EBE**	*CsLOB1* promoter	best score
PthA4	4.95 (91.5%)	10.20 (44.4%)	8.42 (53.8%)	4.53 (100%)
dTALEWTLOB1	5,54 (96.4%)	12,83 (41.6%)	7,55 (70.7%)	5.34 (100%)
dTALEB2A1	7.65 (71.2%)	14.94 (36.5%)	9,65 (56.5%)	5.45 (100%)
dTALELB2A2	3.85 (94.8%)	9.10 (40,1%)	5.65 (64.6%)	3.65 (100%)
dTALELB3A	7.20 (69.4%)	14.49 (34.5%)	9.20 (54.3%)	5.00 (100%)
dTALELB5A	8.95 (58.5%)	14.24 (36.8%)	10.96 (47.8%)	5.24 (100%)

Figure S8 Evolved PthA4-derived TALEs transcriptionally activating the *CsLOB1* promoter and the *Xcc-TALE-trap*. **(a)** *In planta* evolution *CsLOB1* activating TALEs. A PthA4-resembling TALE (dTALEWTLOB1) was modified in its DNA binding domain, resulting in three derivates that do not activate *CsLOB1* (gray font). *In planta* evolution produced four distinct *CsLOB1*-activating TALE proteins (shown in green font) as described previously (Teper and Wang, 2021). TALE repeats are shown as coloured ovals along with their repeat variable diresidues that mediated base preference. Nucleotide sequences at the bottom right show predicted *EBEs* for depicted TALEs in the *CsLOB1* promoter and the two potential target *EBEs* (PthA4-*EBE**, P3213-*EBE**) that are both part of the *Xcc-TALE-trap*. **(b)** TALE-code predicted compatibilty of evolved TALEs and *EBEs* of the *Xcc-TALE-trap*. Scores for the TALE-*EBE* combinations were defined using Target Finder (see Figure 6 for details). Score is also shown as a colour-code with red and blue indicating higher and lower affinity, respectively. TALE repeats differing from PthA4 repeats are highlighted by ovals with dashed lines.
